# Pulmonary insults exacerbate susceptibility to oral *Listeria monocytogenes* infection through the production of IL-10 by NK cells

**DOI:** 10.1371/journal.ppat.1009531

**Published:** 2021-04-20

**Authors:** Nikki Bortell, Elizabeth R. Aguilera, Laurel L. Lenz

**Affiliations:** Department of Immunology and Microbiology, University of Colorado Anschutz Medical Campus, Aurora, CO, United States of America; Duke University School of Medicine, UNITED STATES

## Abstract

Most individuals who consume foods contaminated with the bacterial pathogen *Listeria monocytogenes* (Lm) develop mild symptoms, while others are susceptible to life-threatening systemic infections (listeriosis). Although it is known that the risk of severe disease is increased in certain human populations, including the elderly, it remains unclear why others who consume contaminated food develop listeriosis. Here, we used a murine model to discover that pulmonary coinfections can impair the host’s ability to adequately control and eradicate systemic Lm that cross from the intestines to the bloodstream. We found that the resistance of mice to oral Lm infection was dramatically reduced by coinfection with *Streptococcus pneumoniae* (Spn), a bacterium that colonizes the respiratory tract and can also cause severe infections in the elderly. Exposure to Spn or microbial products, including a recombinant Lm protein (L1S) and lipopolysaccharide (LPS), rendered otherwise resistant hosts susceptible to severe systemic Lm infection. In addition, we show that this increase in susceptibility was dependent on an increase in the production of interleukin-10 (IL-10) from *Ncr1*+ cells, including natural killer (NK) cells. Lastly, the ability of *Ncr1*+ cell derived IL-10 to increase disease susceptibility correlated with a dampening of both myeloid cell accumulation and myeloid cell phagocytic capacity in infected tissues. These data suggest that efforts to minimize inflammation in response to an insult at the respiratory mucosa render the host more susceptible to infections by Lm and possibly other pathogens that access the oral mucosa.

## Author summary

The bacterial pathogen *Listeria monocytogenes* (Lm) causes food-borne infections in humans and animals. Most humans who consume Lm-contaminated foods develop mild symptoms, but in a subset of individuals Lm causes severe systemic infections that are often lethal. Although the factors that predispose individuals to develop severe Lm infection are not well understood, systemic infections require bacteria to disseminate from the intestines to the bloodstream and peripheral tissues. Here we show in a murine model of infection that feeding of Lm alone results in the dissemination of only small numbers of bacteria that are contained and fail to cause symptoms. However, feeding of Lm in mice that also encounter a second infection in the lungs, or have exposure to microbial products in the lungs, results in a severe infection with large numbers of systemic Lm. These lung exposures increase the survival and expansion of Lm that disseminate from the intestines to peripheral tissues by stimulating release of regulatory proteins that dampen the ability of myeloid cells to kill Lm. This study thus reveals how the dampening of inflammation upon microbial exposure at one mucosal tissue can impair the immune response to pathogens entering at a different site and how secondary exposures impact severity of infection in animals that consume Lm-contaminated foods.

## Introduction

The outcome of infections with a given microbial pathogen can vary considerably in different individuals, with some experiencing minimal clinical symptoms and others suffering severe disease or death. This variability in disease phenotype is particularly evident in the ongoing COVID-19 pandemic [1,2]. Whether and how environmental insults or secondary infections may contribute to such phenotypic variations is not known. However, modern clinical molecular diagnostic techniques have revealed that many patients with severe infection-associated disease harbor multiple distinct pathogens [3]. Indeed, it has been estimated that concomitant infections are present in over one sixth of the world’s population [4]. Many such coinfections are associated with more severe disease pathology [4], and some–including HIV and *Mycobacterium tuberculosis* (Mtb) or influenza virus and *Streptococcus pneumoniae* (Spn)–are frequently associated with mortality [5,6]. The mechanistic basis for increased disease severity in these coinfections is not fully-defined, but epidemiologic, clinical, and animal model studies argue that reduction of CD4+ T cells impairs granuloma integrity and macrophage activation to permit increased Mtb replication and dissemination to peripheral organs during HIV:Mtb coinfections [7]. Similarly, skewing of cytokine profiles and myeloid cell responses correlates with increased disease severity and systemic bacterial dissemination during influenza:Spn coinfection [8,9].

*Listeria monocytogenes* (Lm) is a facultative intracellular bacterial pathogen that frequently contaminates processed foods. The severity of infection in individuals who ingest Lm-contaminated foods varies considerably. In some individuals, Lm spreads from the intestines to cause systemic bacteremia and infection of peripheral organs (listeriosis). Listeriosis results in a mortality rate of ~20% [10,11]. The incidence of severe listeriosis in the overall population is low [12,13], but outbreaks of listeriosis occur frequently in association with exposure to Lm-contaminated foods [14]. Longitudinal studies argue that Lm-contaminated foods are consumed numerous times per year [15], but most exposed individuals fail to develop clinically-significant disease symptoms or have self-resolving gastroenteritis. Severe listeriosis typically occurs in a subset of immunocompromised individuals, pregnant women, or the elderly, but these populations do not comprise all individuals who progress to more severe disease. It is unknown if or how specific environmental insults might predispose individuals to severe systemic Lm infections.

Much of our current understanding of listeriosis has benefited from use of a murine model of intravenous (IV) Lm infection. This model has been widely used since the 1960s, and has been paramount to our understanding of immunology and host/pathogen interactions [16,17]. However, this route of infection circumvents the oral/gastrointestinal stage of infection. Several prior investigations have attempted to study Lm infection during oral (PO) inoculation of mice, but like most humans mice fail to develop severe systemic disease when orally exposed to Lm [18,19]. Specifically, while the reported 50% lethal dose (LD_50_) for IV Lm infection range is from 10^4^ to 10^6^ colony forming units (CFUs) depending on the strain of mice infected, oral Lm infection with doses of >10^9^ to 10^11^ CFUs cause only mild disease symptoms in mice [20]. Host resistance to oral Lm appears in part to reflect protection from an intact gut microbiome since mice become more susceptible to local colonization of the gut and Lm-induced gastroenteritis when pre-treated with antibiotics that selectively deplete the resident intestinal microflora [21,22]. However, it is unclear whether such manipulations lead to increased systemic Lm burdens, and antibiotic treatment has not been reported to increase susceptibility to systemic listeriosis in humans [23].

In the current manuscript, we establish that susceptibility of mice to oral Lm is dramatically increased by pulmonary coinfection, and we further identify an immune mechanism that drives more severe systemic listeriosis in this setting. Coinfection selectively increased survival/replication of Lm in peripheral tissues following oral infection and significantly increased the production of the anti-inflammatory cytokine interleukin-10 (IL-10) by Natural Killer (NK) cells. An increase in susceptibility to oral Lm was also induced by intratracheal (IT) instillation of non-replicating immunostimulatory proteins, including a recombinant protein derived from Lm (designated L1S) as well as lipopolysaccharide (LPS). Using a *cre/lox* approach to selectively prevent *Il10* production by *Ncr1*^+^ innate lymphocyte populations, including NK cells, resistance to Lm was restored during coinfections of the lung and gut mucosa. *Ncr1*^+^ production of IL-10 during coinfection correlated with reduced myeloid cell accumulation in Lm infected tissues as well as impairment in the phagocytic capacity of these cells, suggesting that the increased severity of Lm infection in coinfected animals resulted from the triggering of regulatory NK cell IL-10 production to dampen inflammation caused by pulmonary exposure. Thus, coinfection or exposure to agents that trigger pulmonary inflammation can impair host resistance to Lm, and possibly other orally-encountered pathogens.

## Results

### Orally-inoculated mice resist Lm-induced morbidity

**Fig 1:**
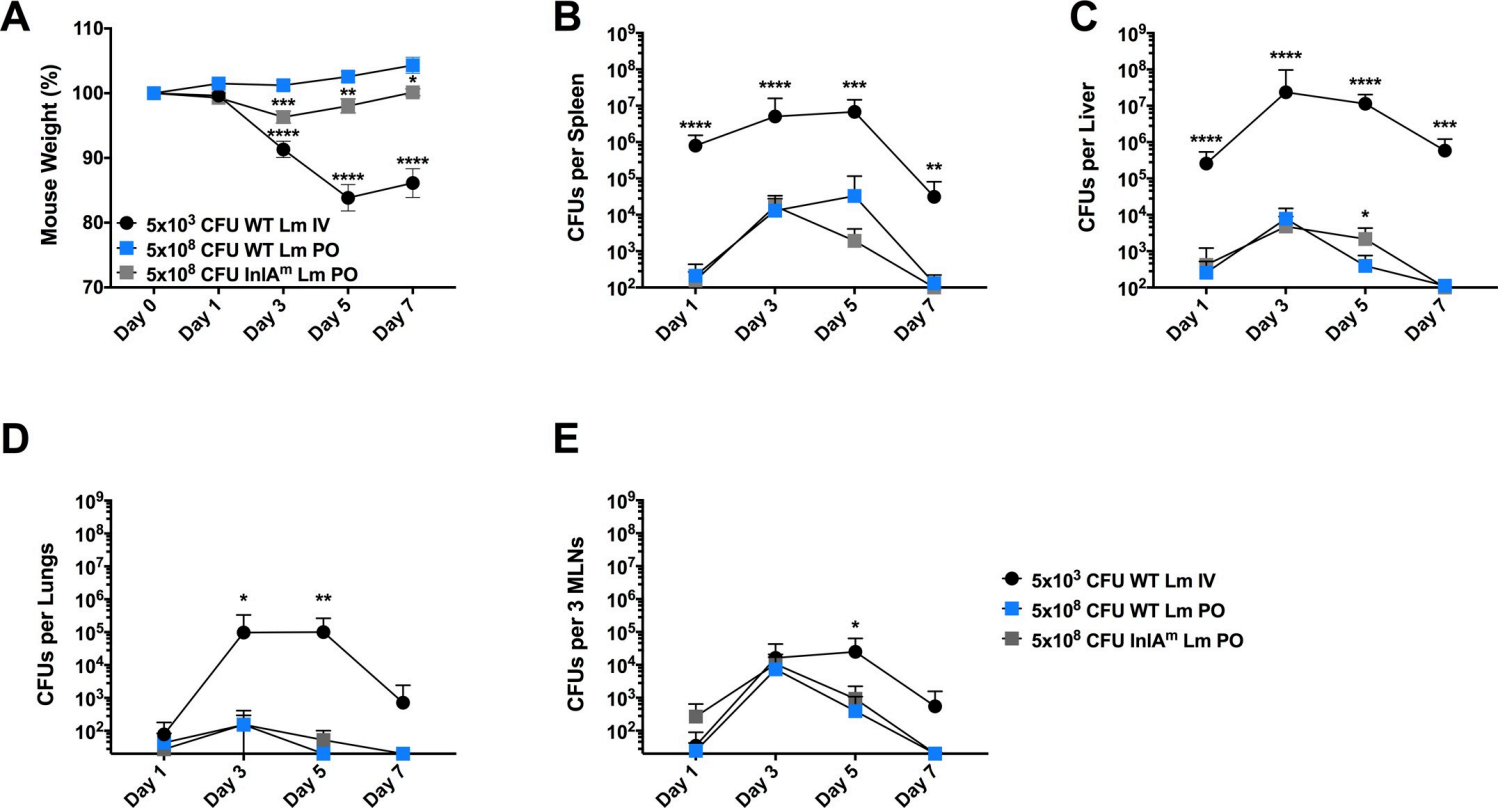
*Orally-inoculated mice resist Lm-induced morbidity*. C57BL/6J (B6) male and female age-matched mice were infected with WT Lm IV, WT Lm PO, or InlA^m^ Lm PO and analyzed over a time course of infection. (A) Mouse weights as a percent % starting weight were determined at each time point. Data are represented as mean ± SEM, Two-way ANOVA, pooled from 3 experiments, with 8–15 mice per group, *p<0.05, **p<0.002, ***p<0.0002, ****p<0.0001 between WT Lm PO and indicated groups. In addition, CFUs were determined for each infection at the given time point in the following tissues (B) spleen, (C) liver, (D) lungs, (E) mesenteric lymph nodes (MLNs). Data are represented as mean ± SD, Mann-Whitney t-test, pooled from 3 experiments, with 8–15 mice per group, *p<0.05, **p<0.002, ***p<0.0002, ****p<0.0001 between WT Lm PO and indicated groups.

To investigate the kinetics and severity of *Listeria monocytogenes* (Lm) infection upon oral inoculation, C57BL/6J (B6) mice were challenged with sublethal doses of intravenous (IV) or peroral (PO) Lm. Co-housed sex and age matched mice were divided into groups and challenged with 5x10^3^ CFUs strain 10403S (WT) Lm IV, 5x10^8^ CFUs strain 10403S (WT) Lm PO, or with 5x10^8^ CFUs of a strain of Lm engineered to express the murinized form of internalin A (InlA^m^; 10403S background). To maintain intestinal homeostasis, no antibiotic pretreatment, gastric pH adjustment, or starvation methods were used before oral inoculation of Lm. We evaluated overall morbidity (as measured by percent weight loss) as well as bacterial burdens (as measured by colony forming units, CFUs) in peripheral organs over the first seven days of the Lm infections. Despite inoculation with 5-log fewer bacteria, IV Lm infection resulted in significantly higher morbidity and bacterial burdens in spleens, livers, and lungs when compared to PO Lm infection (Fig 1A–1D). Morbidity was limited during oral Lm infection, yet PO-administered Lm successfully disseminated to peripheral tissues and the kinetics of bacterial detection in spleens, livers, lungs, and mesenteric lymph nodes (MLNs) were similar regardless of the route of infection (Fig 1A–1E). Namely, following IV or PO infection, Lm numbers peaked at 3 to 5 days post infection (dpi), and the mice began to show reduced peripheral burdens by 7 dpi (Fig 1B–1E). Previous studies have reported that WT Lm has a reduced ability to invade mouse intestinal epithelial cells due to the poor binding capacity between the Lm internalin protein (InlA) and mouse intestinal E-cadherin [24]. We thus evaluated oral infection with an isogenic Lm strain expressing a “murinized” InlA^m^ allele that binds murine E-cadherin with higher affinity [25]. Our results show that the kinetics and magnitude of Lm burdens in peripheral tissues were indistinguishable following oral inoculation with Lm strains expressing WT or InlA^m^ (Fig 1A–1E). These data indicate that the failure of orally-inoculated mice to develop disease symptoms (e.g. weight loss) likely reflects the low peripheral Lm burdens following oral infection, which are irrespective of the bacterial InlA allele. Further, these data show that Lm disseminates into the bloodstream following oral Lm but minimally expands in peripheral tissues. Together, these findings suggest that the immune response is more effective at containing Lm entering via the oral versus IV route.

### Pulmonary Spn coinfection increases susceptibility to oral Lm

**Fig 2:**
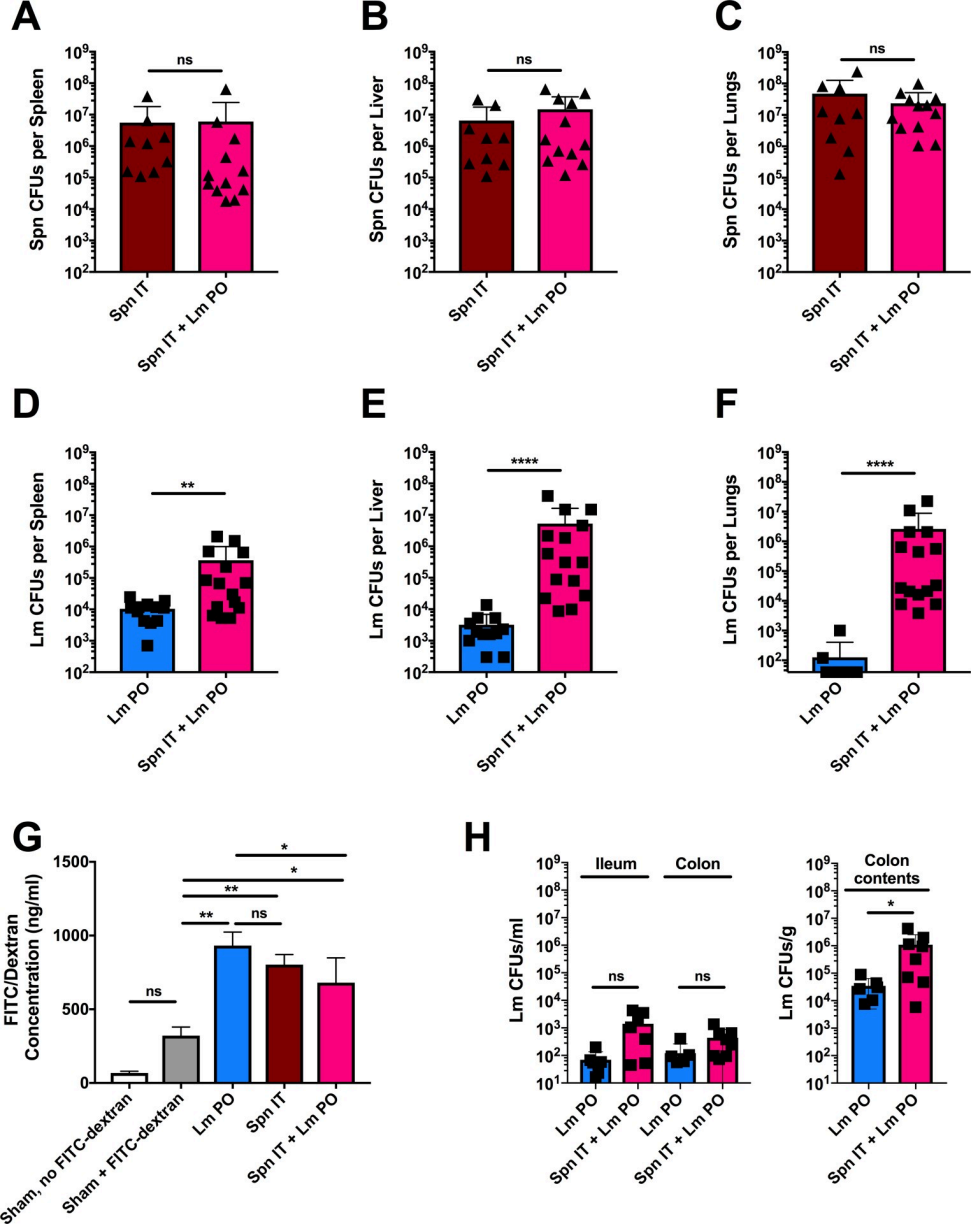
*Pulmonary Spn coinfection increases susceptibility to oral Lm*. C57BL/6J (B6) male and female age-matched mice were infected with sham control, Lm PO, Spn IT, or Spn IT + Lm PO, and CFUs were determined 3 dpi. Spn burdens were enumerated from (A) spleens, (B) livers, and (C) lungs, and Lm burdens were enumerated from (D) spleen, (E) livers, and (F) lungs. (A-F) Data are represented as mean ± SD, Mann-Whitney t-test, pooled from 3 experiments, with 9–15 mice per group, **p<0.002, ****p<0.0001 between indicated groups. B6 male and female age-matched mice were infected with sham control, Lm PO, or Spn IT + Lm PO, and gut permeability and CFUs were determined 1 dpi. (G) FITC-dextran was orally gavaged 4 hour before sacrifice, and upon sacrifice, blood was collected by cardiac puncture. Serum was measured for fluorescence, and the concentration was determined using a standard curve. Data represent mean ± SD, One-way ANOVA, pooled from 2 experiments, 4–8 mice per group, *p<0.05, **p<0.002 between indicated groups. (H) Intestinal Lm CFUs were measured from the ileum, colon, and in the colon contents. Data represent mean ± SD, Mann-Whitney t-test, pooled from 2 experiments, 6–8 mice per group, *p<0.05 between indicated groups.

Secondary or concurrent infections can alter the development of protective immune responses and increase the severity of pneumonias and invasive systemic infections by pathogens such as *Streptococcus pneumoniae* (Spn), *Staphylococcus aureus*, and *Mycobacterium tuberculosis* [9,26,27]. We therefore considered if a secondary respiratory infection might promote increased systemic Lm burdens following oral exposure. To test this, we established a model of concurrent pulmonary Spn infection and oral administration of Lm. Briefly, anesthetized mice received 50μl containing 10^5^ CFUs Spn intratracheally (IT), followed by oral gavage with 200μl containing 5x10^8^ CFUs WT Lm. Sham-infected mice received 50μl PBS IT + 200μl PBS PO, and singly infected mice received the appropriate infection with the alternative PBS control inoculation. At 3 dpi, tissues were harvested and plated on selective media to enumerate streptomycin-resistant, neomycin-sensitive Lm and neomycin-resistant, streptomycin-sensitive Spn. Quantitation of bacterial CFUs revealed that tissue burdens of IT-inoculated Spn were not significantly altered by PO coinfection with Lm (Fig 2A–2C). In contrast, Spn coinfection increased burdens of orally administered Lm by 10^2^ to over 10^4^ fold in all tested tissues (spleens, livers, and lungs) (Fig 2D–2F). To ensure the fidelity of our oral and IT treatments, we used Trypan Blue to confirm minimal aspiration of orally-gavaged Trypan Blue into the lungs, or of IT-instillation of Trypan Blue into the stomach (S1 Fig). To further address whether these dramatic increases in peripheral Lm burdens might reflect an increase in the permeability of the intestines at an earlier time point, groups of PO Lm and coinfected mice were orally treated with FITC-dextran four hours prior to sacrifice. Quantitation of serum fluorescence at 1 dpi revealed that compared to sham infected mice, Lm PO infection, Spn IT infection, and coinfection, increased concentrations of FITC-dextran in the blood (Fig 2G). However, a similar or greater accumulation of FITC-dextran was seen during oral Lm infection alone–despite substantially lower peripheral bacterial burdens. These data indicate that egress of Lm to peripheral tissues correlates with an increase in intestinal leakage, but that the increase in peripheral Lm burdens during coinfection was not caused by an increase in intestinal leakage.

We also investigated the possibility that coinfection might increase Lm burdens in the gut. Here, mice were orally infected with Lm in the presence or absence of pulmonary Spn coinfection. At 1 dpi, Lm burdens in ileal and colon tissues, as well as in the colon contents, were enumerated. Lm numbers in the colon contents were found to be increased by ~ 10-fold in the coinfected animals (Fig 2H). However, this does not likely explain the increase in peripheral Lm burdens, since the number of Lm closely associated with the ileal and colonic tissues were not significantly increased in coinfected mice (Fig 2H). Together with the results above, these data indicate that Spn coinfection increases the survival/expansion of Lm in peripheral tissues and suggest that the majority of this increase occurs following dissemination of the oral Lm bacteria from the gut into the periphery.

### Susceptibility to oral Lm correlates with IL-10 production by NK cells

**Fig 3:**
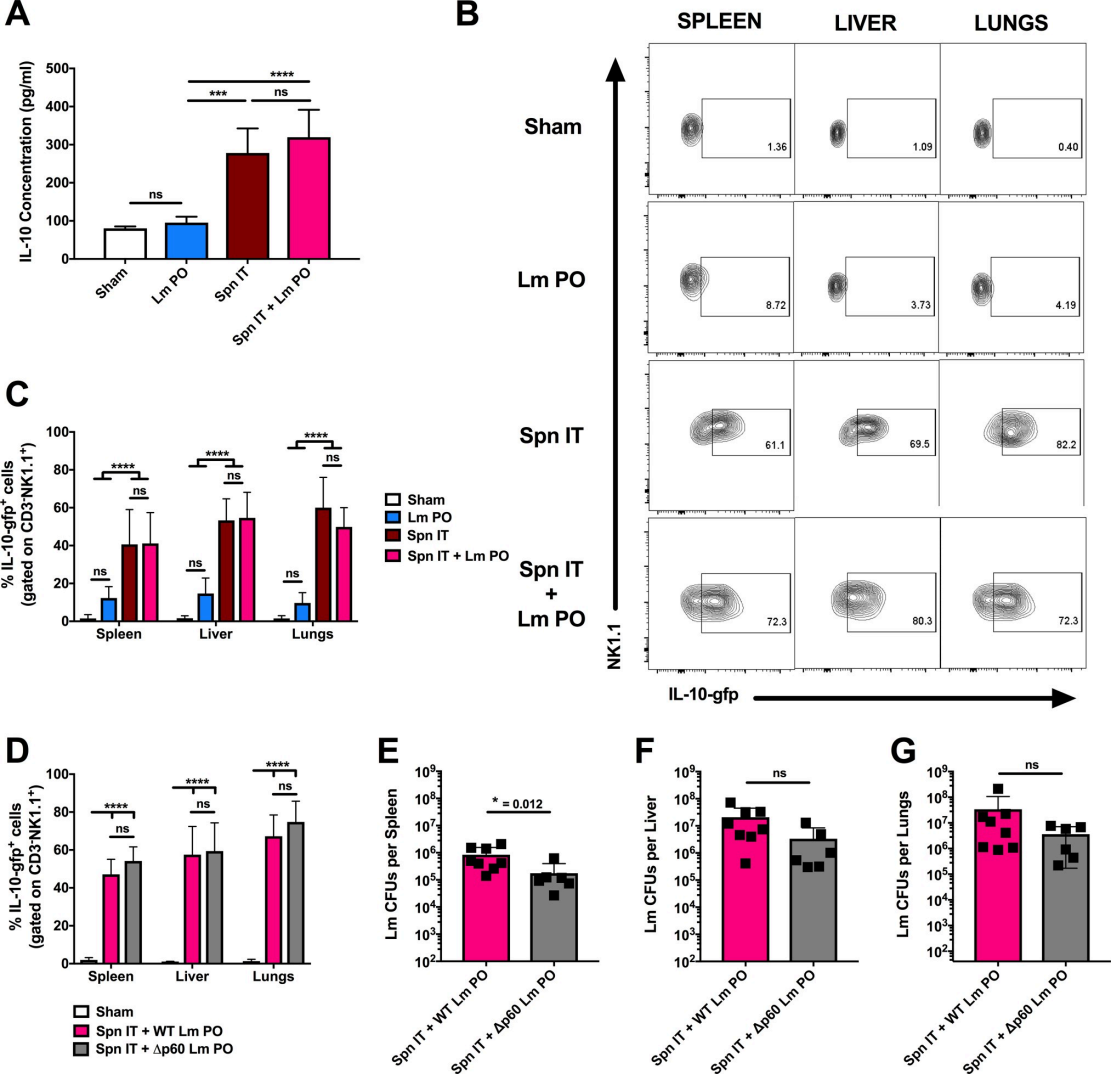
*Susceptibility to oral Lm correlates with IL-10 production by NK cells*. B6.*tiger* (*Il10*-gfp reporter) male and female age-matched mice were infected with sham control, Lm PO, Spn IT, or Spn IT + Lm PO and harvested at 3 dpi. (A) Upon sacrifice, blood was collected by cardiac puncture, and serum IL-10 was measured by ELISA. Data represent mean ± SD, One-way ANOVA, pooled from 2 experiments, 4–6 mice per group, ***p<0.0002, ****p<0.0001 between indicated groups. (B) Representative flow plots of reporter staining in CD3^-^NK1.1^+^ NK cells in indicated tissues. (C) Quantitation of percent CD3^-^NK1.1^+^ NK cells that stained positive for IL-10-gfp^+^ in spleens, livers, and lungs. Data represent mean ± SD, Two-way ANOVA, pooled from 3 experiments, 6–12 mice per group, ****p<0.0001 between indicated groups. B6.*tiger* (*Il10*-gfp reporter) male and female age-matched mice were infected with sham control, Spn IT + WT Lm PO, or Spn IT + Δp60 Lm Lm PO and harvested at 3 dpi for flow analysis and CFU enumeration. (D) Quantitation of percent CD3^-^NK1.1^+^ NK cells that stained positive for IL-10-gfp^+^ in spleens, livers, and lungs. Data represent mean ± SD, Two-way ANOVA, pooled from 2 experiments, 6–8 mice per group, ****p<0.0001 between indicated groups. Lm burdens were enumerated from (E) spleens, (F) livers, (G) lungs. Data represent mean ± SD, Mann-Whitney t-test, pooled from 2 experiments, 6–8 mice per group, *p<0.05.

To investigate whether Spn coinfection might suppress innate immune responses to oral Lm, we quantified serum concentrations of 10 cytokines implicated in the initiation and regulation of inflammatory response using a commercial array panel. This analysis indicated that serum concentrations for all tested cytokines remained indistinguishable between sham-infected mice and those given oral Lm alone at 3dpi (S2A–S2J Fig). By comparison, Spn single infection as well as coinfection significantly increased serum IL-10, IL-12p70, IL-1β, IL-6, KC/GRO, and TNFα, while IFNγ concentrations were moderately, but not significantly, increased during coinfection, and IL-4 concentrations were only increased when mice were exposed to pulmonary Spn alone. To further identify whether other chemokines besides KC/GRO were impacted by infection, we also examined the production of CCL2 and CCL3 by ELISA. Serum levels of both of these chemokines were unaffected by Lm PO infection, but were significantly increased during Spn single infection as well as during coinfection (S2K–S2L Fig). While many cytokines and chemokines were induced upon Spn infection and coinfection, none were induced by Lm PO infection alone, and we therefore hypothesized that uncontrolled peripheral Lm burdens during coinfection may be due to a dampening of the immune response. Here we focused on IL-10, a cytokine with a variety of potent anti-inflammatory effects that is known to increase host susceptibility during systemic Lm infection [28,29,30]. We verified, using standard ELISA techniques, that serum concentrations of IL-10 were not significantly elevated following oral exposure to Lm alone, but that serum IL-10 was significantly increased following pulmonary exposure to Spn and Spn IT + Lm PO coinfection (Fig 3A). To identify the cellular sources of IL-10, we evaluated gfp expression using tiger (*Il10-gfp*) reporter mice. Tiger mice were treated with PBS as a sham control, infected with Lm PO, Spn IT, or coinfected, then harvested at 3 dpi. Gating strategies to evaluate gfp staining by splenic B, T, NKT, NK, and other populations are shown in S3 Fig, and representative IL-10-gfp^+^ staining for these populations are shown in S4 Fig. In addition, we analyzed the production of IL-10 by inflammatory monocytes and neutrophils (gating strategies are shown in S5 Fig, and representative IL-10-gfp^+^ staining for these populations are shown in S6 Fig). Taken together these data show that NK1.1^+^ cells are the predominant population that was found to stain positive for gfp.

We thus further characterized IL-10-gfp^+^ staining in spleen, liver, and lungs from sham, oral Lm, pulmonary Spn and coinfected mice. IL-10 reporter expression was negligible in sham infected mice or mice given oral Lm infection alone, while NK cells shifted to stain positive for IL-10-gfp^+^ in mice infected with Spn IT or coinfected with Spn IT + Lm PO (Fig 3B). Quantification of these staining data confirmed that the proportion of NK cells producing IL-10 during oral Lm infection did not significantly increase in the spleen, liver, and lungs, while both Spn IT and coinfection significantly increased the proportion of IL-10-gfp^+^ NK cells (Fig 3C). These data further showed that the proportion of NK cells staining positive for the IL-10 reporter was comparable in Spn infected versus coinfected mice (Fig 3C). Importantly, we observed no differences in bacterial burdens or in reporter expression between male and female mice (S7 Fig). Hence, the low peripheral bacterial burdens following oral Lm infection alone correlated with a failure to elicit IL-10 production by NK cells, whereas increased NK cell IL-10 and serum IL-10 were observed to correlate with the increased systemic Lm burdens following Spn coinfection.

During IV infection, Lm secretes a virulence protein called p60, which permits increased replication of Lm in peripheral tissues. Our lab has previously shown that during IV infection with a strain of Lm that is deficient in the expression of p60 (Δp60 Lm), bacterial burdens are reduced [31], and that the reduction in peripheral bacterial burdens upon infection with Δp60 Lm IV correlated with a reduction in NK cell production of IL-10 [32]. We thus considered whether Lm expression of p60 might contribute to the increased IL-10 production observed during Spn IT + Lm PO coinfection. Remarkably, we observed no difference in the percent of NK cells producing IL-10 when *Il10-gfp* reporter mice were coinfected with Spn IT + WT Lm PO versus Spn IT + Δp60 Lm PO (Fig 3D). Furthermore, in these coinfected mice, the absence of p60 expression had only a minimal impact on Lm burdens in the spleens, with no significant difference in the CFUs of Δp60 versus WT Lm recovered from livers or lungs (Fig 3E–3G). These data suggest that, unlike during IV Lm infection, NK cell IL-10 responses in coinfected mice are completely independent of Lm p60 expression. Further, these data argue that in the coinfected mice, the expression of p60 is largely dispensable for systemic Lm replication. Importantly, we observed no differences in Spn CFUs when mice were coinfected with WT versus Δp60 Lm (S8A–S8C Fig). Overall, these data suggest that the production of IL-10 in Spn IT + Lm PO coinfected mice may be driven by Spn and support the hypothesis that NK cell IL-10 production, which is not seen during oral infection by Lm alone, may directly contribute to the increased systemic replication of Lm in coinfected mice.

### Exacerbated Lm burdens require the production of IL-10 by Ncr1^+^ cells

**Fig 4:**
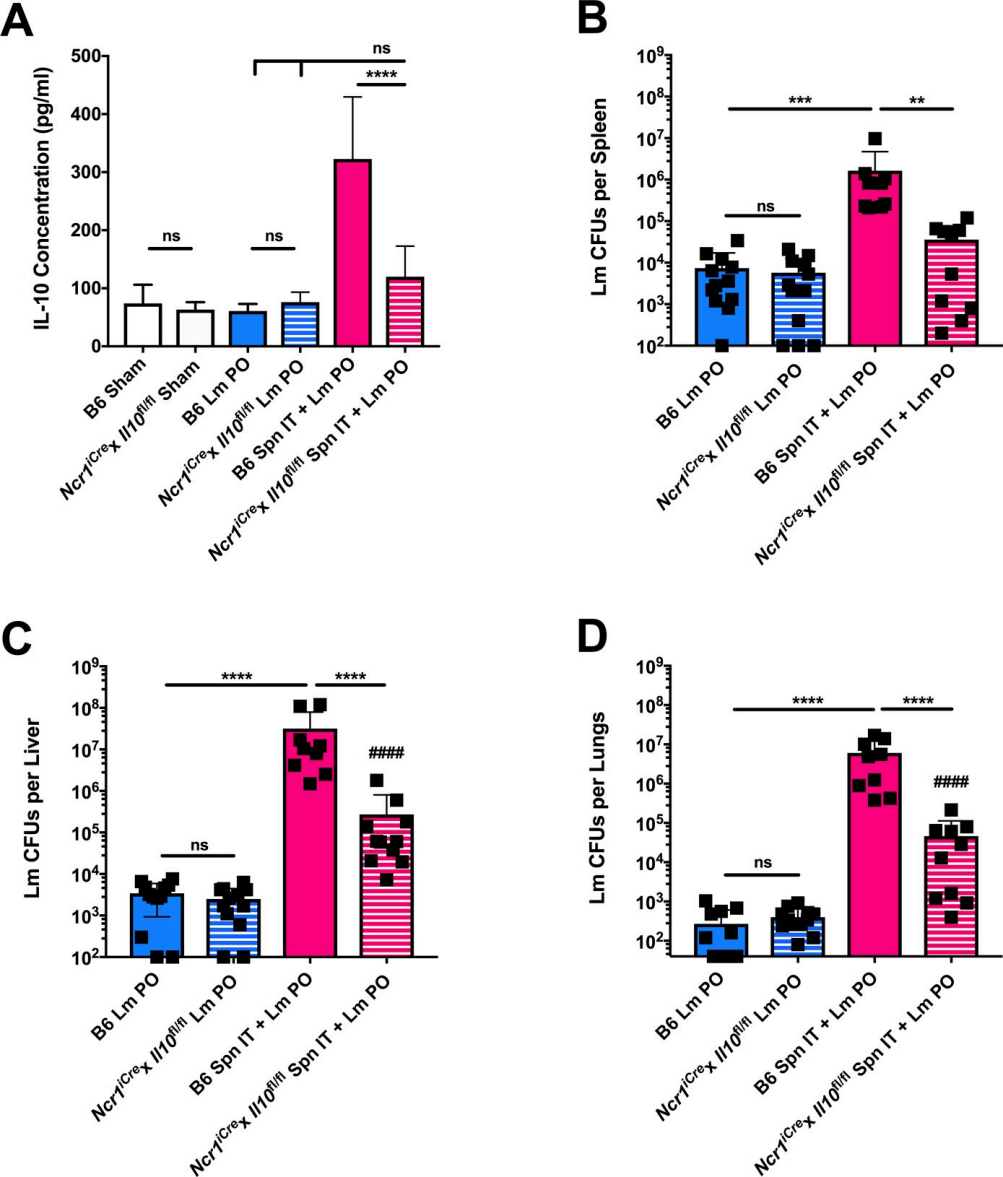
*Exacerbated Lm burdens require the production of IL-10 by Ncr1*^*+*^ *cells*. C57BL/6J (B6) and *Ncr1*^*iCre*^x*Il10*^fl/fl^ male and female age-matched mice were infected with sham control, Lm PO, or Spn IT + Lm PO and harvested at 3 dpi. (A) Upon sacrifice, blood was collected by cardiac puncture, and serum IL-10 was measured by ELISA. Data represent mean ± SEM, One-way ANOVA, pooled from 2 experiments, 6 mice per group, ****p<0.0001 between indicated groups. Lm burdens were enumerated from (B) spleens, (C) livers, (D) lungs. Data represent mean ± SD, Mann-Whitney t-test, pooled from 3 experiments, 9–12 mice per group. **p<0.002, ***p<0.0002, ****p<0.0001 between indicated groups, ####p<0.0001 between *Ncr1*^*iCre*^x*Il10*^fl/f^ coinfected mice and B6 Lm PO infected mice.

To assess the impact of NK cell production of IL-10 more directly in coinfected mice, we used an *Ncr1*^*iCre*^x*Il10*^fl/fl^ mouse strain in which *Il10* expression is selectively lost in *Ncr1*-expressing cells. *Ncr1* expression is largely restricted to NK cells, though expression can be seen in certain other innate lymphoid cell types [33,34]. Age and sex-matched B6 and *Ncr1*^*iCre*^x*Il10*^fl/fl^ mice were infected with Lm PO or coinfected with Spn IT + Lm PO and harvested at 3 dpi. When serum IL-10 was measured by ELISA, we confirmed that the selective loss of *Il10* in *Ncr1*^+^ cells was sufficient to significantly reduce serum IL-10 concentrations (Fig 4A). Consistent with this reduction in IL-10, Lm burdens in spleens, livers, and lungs of coinfected *Ncr1*^*iCre*^x*Il10*^fl/fl^ mice were significantly lower than in coinfected B6 mice (Fig 4B–4D). Importantly, the *Ncr1*^*iCre*^x*Il10*^fl/fl^ mice were not inherently better at restricting access of oral Lm to the bloodstream or Lm replication in peripheral tissues in the absence of Spn coinfection (Fig 4B–4D). These data confirm that, to a large extent, the dramatic increase in peripheral Lm burdens during coinfection in B6 mice is dependent on the induction of IL-10 production by *Ncr1*^+^ (presumably NK) cells. However, the fact that coinfection still sufficed to modestly increase Lm burdens in *Ncr1*^*iCre*^x*Il10*^fl/fl^ mice compared to oral Lm alone (Fig 4B–4D) argues that there may also be additional NK cell-dependent or independent mechanisms that contribute to this phenomenon.

### IL-10 from Ncr1+ cells limits myeloid cell accumulation and phagocytic capacity during coinfection

**Fig 5:**
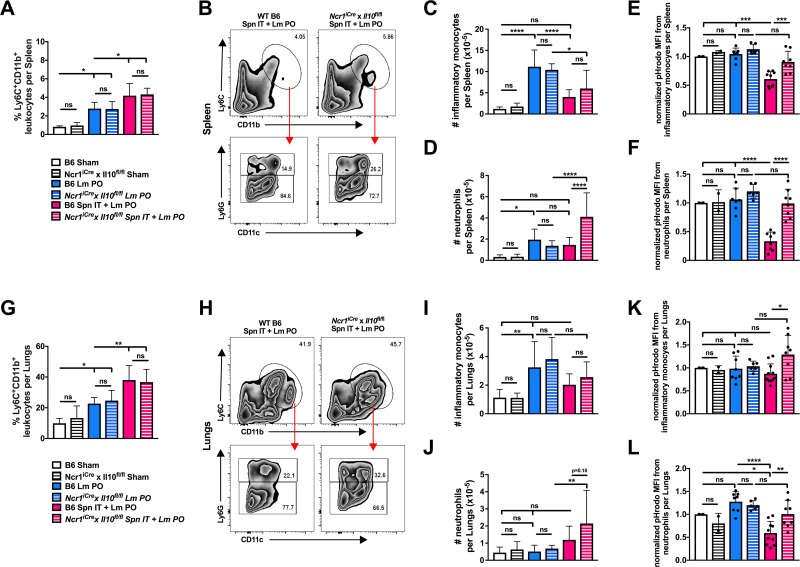
*IL-10 from Ncr1+ cells limits myeloid cell accumulation and phagocytic capacity during coinfection*. C57BL/6J (B6) and *Ncr1*^*iCre*^x*Il10*^fl/fl^ male and female age-matched mice were infected with sham control, Lm PO, or Spn IT + Lm PO and harvested at 3 dpi. Quantitation of percent CD45.2^+^Ly6C^+^CD11b^+^ leukocytes in (A) spleens and (G) lungs. Data represent mean ± SD, One-way ANOVA, pooled from 3 experiments, 6–12 mice per group, *p<0.05, **p<0.002 between indicated groups. Representative flow plots of Ly6G^+^ neutrophils and Ly6G^-^CD11c^l^° inflammatory monocytes in B6 and *Ncr1*^*iCre*^x*Il10*^fl/fl^ coinfected mice in (B) spleens and (H) lungs. Quantitation of total number of CD45.2^+^Ly6C^+^CD11b^+^ cells that are Ly6G^-^CD11c^l^° inflammatory monocytes in spleens (C) or Ly6G^+^ neutrophils in spleens (D) or that are Ly6G^-^CD11c^l^° inflammatory monocytes in lungs (I) or Ly6G^+^ neutrophils in lungs (J). Data represent mean ± SD, One-way ANOVA, pooled from 3 experiments, 6–12 mice per group, *p<0.05, **p<0.002, ****p<0.0001 between indicated groups. pHrodo *S*. *aureus* Bioparticles were incubated for 1 hr with cells isolated from spleens and lungs. Normalized mean fluorescent intensity (MFI) of pHrodo positive inflammatory monocytes and neutrophils was determined by flow cytometry. pHrodo MFI from inflammatory monocytes in spleens (E), neutrophils in spleens (F), inflammatory monocytes in lungs (K), and neutrophils in lungs (L). Data represent mean ± SD, One-way ANOVA, pooled from 2 experiments, 8–9 mice per group, *p<0.05, **p<0.002, ***p<0.0002, ****p<0.0001 between indicated groups.

Myeloid cells are known to be critical in the early control of Lm burdens during systemic IV infection [35,36]. In addition, IL-10 has been shown to suppress M1-type myeloid cell activation and decrease myeloid cell infiltration into tissues [37,38,39,40]. We therefore wanted to determine whether inflammatory monocytes or neutrophils were impacted by coinfection and the induction of NK cell IL-10. Gating strategies to identify inflammatory monocytes and neutrophils by flow cytometry are shown in S5 Fig. B6 and *Ncr1*^*iCre*^x*Il10*^fl/fl^ mice were sham infected, infected with Lm PO, or coinfected, and myeloid cells were examined in spleens, livers, and lungs by flow cytometry at 3 dpi. In both strains of mice, the proportion of Ly6C^+^CD11b^+^ inflammatory myeloid cells in the spleen and lungs were increased by oral Lm infection and even further increased by coinfection (Fig 5A and 5G). Additionally, the accumulating cells were shown to be a mixture of Ly6G^+^ neutrophils and Ly6G^-^CD11c^l^° inflammatory monocytes as shown by representative gating in coinfected B6 mice and *Ncr1*^*iCre*^x*Il10*^fl/fl^ mice (Fig 5B and 5H). In regard to total cell number, in both spleens and lungs, there was no difference in the number of inflammatory monocytes or neutrophils between B6 mice and *Ncr1*^*iCre*^x*Il10*^fl/fl^ mice in either sham infected animals, or oral Lm infected animals (Fig 5C, 5D, 5I and 5J), suggesting that the ability of *Ncr1*+ cells to produce IL-10 has no bearing on myeloid cell numbers under steady state conditions or in the context of oral Lm infection alone. Interestingly, in B6 mice, oral Lm infection significantly increased the number of inflammatory monocytes and neutrophils in the spleens, and the number of inflammatory monocytes in the lungs, compared to sham infection (Fig 5C, 5D, 5I and 5J). In contrast, coinfection in B6 mice significantly decreased the number of inflammatory monocytes in the spleens when compared to oral infection alone (Fig 5C). In addition, coinfection in B6 mice did not significantly alter the number of inflammatory monocytes or neutrophils in the spleens or lungs when compared to sham infected animals (Fig 5C, 5D, 5I and 5J). However, upon coinfection, the selective deletion of Il10 in *Ncr1*+ cells significantly increased the number of neutrophils when compared to B6 coinfected animals in both spleens and lungs (Fig 5D and 5J). Data from livers are shown in S9 Fig, where a similar increase in the number of neutrophils is seen in *Ncr1*^*iCre*^x*Il10*^fl/fl^ mice upon coinfection when compared to Lm PO infected mice. These results suggest that coinfection blunts the infiltration of inflammatory monocytes into spleens, and that the production of IL-10 by *Ncr1*+ cells suppresses the accumulation of neutrophils in peripheral tissues. In this context, the lack of accumulation of these cells may promote the increase in bacterial burdens seen during coinfection.

IL-10 has been shown to inhibit phagosomal maturation in macrophages infected with Mtb [41]. We therefore wanted to examine whether the production of IL-10 by Ncr1+ cells during coinfection similarly impacted the phagocytic capacity of inflammatory monocytes and neutrophils. To address this, we incubated splenic and pulmonary cells with fluorescence-labeled *Staphylococcus aureus* Bioparticles for 1 hr at 37°C, where the bioparticles are labeled with a pH-sensitive dye (pHrodo) that only fluoresces at an acidic pH. Flow cytometry allowed for the evaluation of particle uptake into matured, acidified compartments in both inflammatory monocytes and neutrophils. Here we show that the proportion of inflammatory monocytes in the lungs, and neutrophils in the spleens and lungs, were not significantly altered by either mode of infection or by mouse phenotype (S10A, S10C, S10E and S10G Fig). Although the proportion of inflammatory monocytes and neutrophils that stained positive for pHrodo were in general similar across infections and between B6 and *Ncr1*^*iCre*^x*Il10*^fl/fl^ mice, the mean fluorescent intensity (MFI) for pHrodo staining in these cells was significantly altered by coinfection. Representative shifts in MFI of pHrodo staining can be seen in S10B, S10D, S10F and S10H Fig. As seen in Fig 5E, 5F and 5L, the MFI of pHrodo staining in inflammatory monocytes and neutrophils in spleens, and in neutrophils in the lungs, was significantly decreased upon coinfection compared to oral Lm infection or sham infection in B6 mice. Importantly, Lm PO infection did not alter pHrodo staining when compared to sham infected mice, in either B6 or *Ncr1*^*iCre*^x*Il10*^fl/fl^ mice. However, pHrodo MFI staining in inflammatory monocytes and neutrophils in both spleens and lungs were significantly increased in coinfected *Ncr1*^*iCre*^x*Il10*^fl/fl^ mice when compared to coinfected B6 mice, and indeed, levels were restored to those seen in both sham and Lm PO infected mice (Fig 5E, 5F, 5K and 5L). These data indicate that on a per cell basis, inflammatory monocytes and neutrophils have a reduced ability to phagocytose bacteria into compartments that are maturing when in the presence of *Ncr1*+ derived IL-10. Overall, the production of IL-10 by *Ncr1*+ cells during coinfection limits inflammatory monocyte and neutrophil infiltration into infected tissues, and blunts the phagocytic capacity of these cells, which may contribute to the increase in peripheral Lm burdens seen during coinfection.

### Pulmonary instillation of a bacterial protein suffices to stimulate NK cell IL-10 production and increase peripheral burdens of orally-inoculated Lm

**Fig 6:**
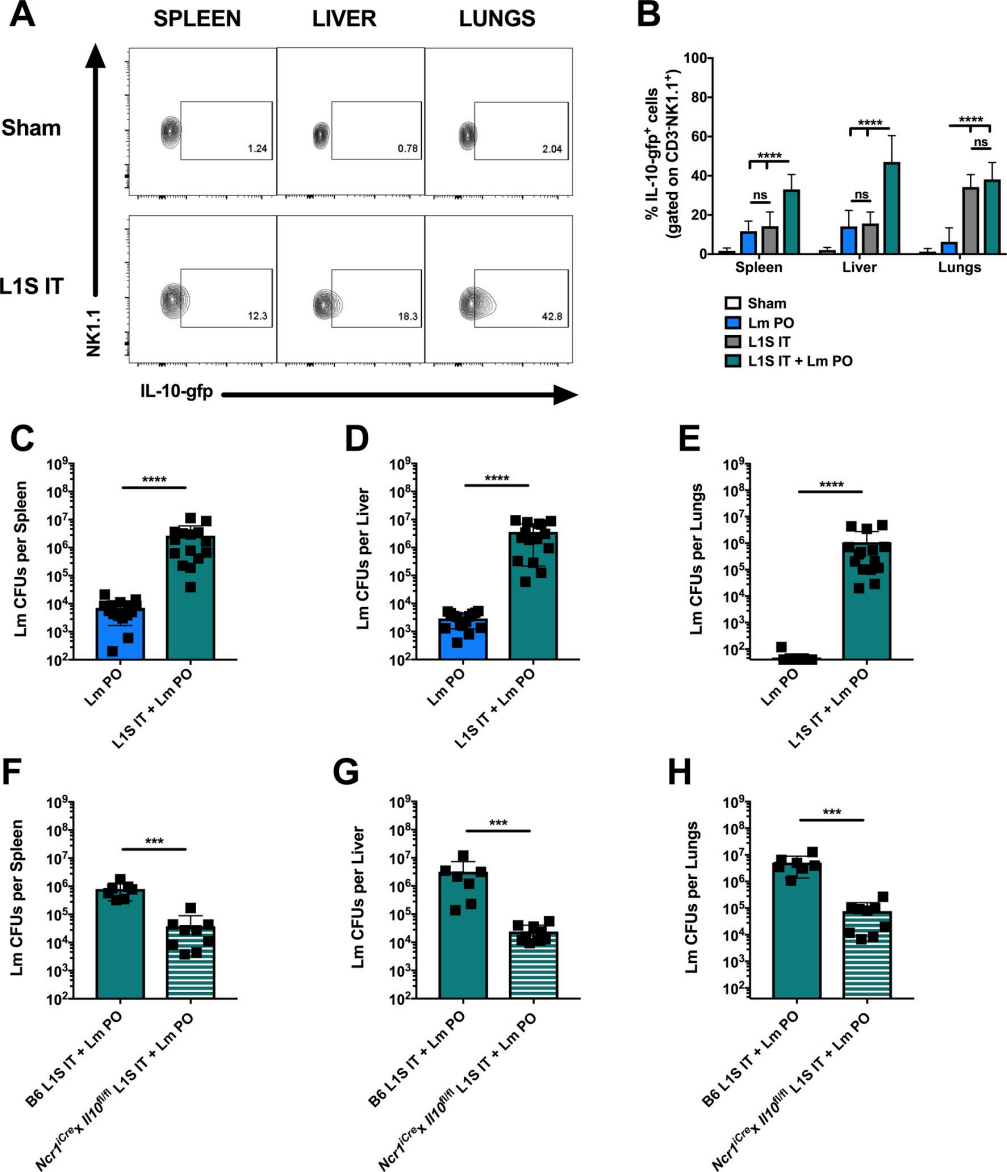
*Pulmonary instillation of a bacterial protein suffices to stimulate NK cell IL-10 production and increase peripheral burdens of orally-inoculated Lm*. B6.*tiger* (*Il10*-gfp reporter) male and female age-matched mice were infected with sham control or treated with 100ug L1S intratracheally (IT) and harvested at 3 dpi. (A) Representative flow plots of reporter staining in CD3^-^NK1.1^+^ NK cells in indicated tissues. B6.*tiger* (*Il10*-gfp reporter) male and female age-matched mice were infected with sham control, Lm PO, L1S IT, or L1S IT + Lm PO and harvested at 3 dpi. (B) Quantitation of percent CD3^-^NK1.1^+^ NK cells that stained positive for IL-10-gfp^+^ in spleens, livers, and lungs. Data represent mean ± SD, Two-way ANOVA, pooled from 2 experiments, 8 mice per group, ****p<0.0001 between indicated groups. Lm burdens were enumerated from (C) spleens, (D) livers, (E) lungs. Data represent mean ± SD, Mann-Whitney t-test, pooled from 3 experiments, 15 mice per group, ****p<0.0001. C57BL/6J (B6) and *Ncr1*^*iCre*^x*Il10*^fl/fl^ male and female age-matched mice were infected with L1S IT + Lm PO and harvested at 3 dpi. Lm burdens were enumerated from (F) spleens, (G) livers, (H) lungs. Data represent mean ± SD, Mann-Whitney t-test, pooled from 2 experiments, 7–9 mice per group, ***p<0.0002.

The data above indicate that Spn infection drives the production of IL-10 by NK cells to increase susceptibility to oral Lm infection. However, as shown in Fig 2A–2C, Spn burdens during infection were not restricted to the lungs. We therefore wanted to determine whether localized stimuli could similarly increase susceptibility to oral Lm infection in a manner that was dependent on NK cell production of IL-10. Our lab has previously used a truncated recombinant derivative of the Lm p60 virulence protein (designated L1S) to stimulate the production of IL-10 by NK cells in an *in vitro* model system [32]. Here we established an in vivo model of IT instillation of L1S into tiger mice, and evaluated IL-10 production by NK cells at 3 dp instillation. We found that L1S instillation (100μg in 50μl) potently induced IL-10-gfp^+^ NK cells in the lungs of instilled mice, with more moderate increases in IL-10-gfp^+^ splenic and hepatic NK cell populations (Fig 6A).

Using this model of L1S instillation, we next treated *Il10-gfp* mice IT with PBS as a sham control or with L1S protein diluted in PBS, then mock infected or infected the mice PO with 5 x10^8^ WT Lm. At 3 dpi, mice were harvested, and reporter expression in immune cells was quantified. S11 Fig shows representative flow staining, which verified NK cells as the predominate cell population expressing IL-10. When compared to oral Lm alone, L1S IT treatment did not significantly alter the proportion of NK cells making IL-10 in either the spleen or the liver (Fig 6B). However, compared to Lm PO, both L1S IT and L1S IT + Lm PO significantly increased NK cell IL-10 in the lungs. In addition, when given together with oral Lm, L1S treatment further increased the proportion of NK cells making IL-10 in spleens and livers (Fig 6B). Consistent with its ability to induce or amplify NK cell IL-10-gfp^+^ staining, L1S treatment also caused a significant increase in peripheral Lm burdens in the spleens, livers, and lungs of cotreated mice (Fig 6C-6E). Experiments in the *Ncr1*^*iCre*^x*Il10*^fl/fl^ mice further showed that the L1S treatment failed to increase Lm burdens following PO Lm inoculation (Fig 6F-6H). Thus, pulmonary treatment with L1S was sufficient to promote NK cell production of IL-10 and increase susceptibility to oral Lm in a manner that required IL-10 production by *Ncr1*^+^ cells. Overall, these data show that an alternative driver of NK cell IL-10 is sufficient to increase peripheral burdens upon oral infection with Lm.

These data led us to question whether *any* pulmonary stimuli could increase susceptibility to oral Lm infection. To determine whether other pulmonary stimuli could increase peripheral Lm burdens, we instilled 10^6^ heat-killed (HK) Spn into the lungs of mice, followed by oral Lm infection. As seen in S12A–S12C Fig, HK Spn significantly increased Lm burdens in spleens, livers, and lungs at 3dpi when compared to oral Lm infection alone. We next instilled three different TLR agonists, including LPS, poly:IC, and Pam3CSK4 and examined Lm burdens upon oral infection. Interestingly, only LPS increased peripheral Lm burdens (S12D–S12F Fig). In addition, both HK Spn as well as LPS increased peripheral Lm burdens in a manner that was dependent on *Ncr1*+ cell production of IL-10. As seen in S12G–S12I Fig, during co-treatment, Lm burdens were significantly reduced in *Ncr1*^*iCre*^x*Il10*^fl/fl^ mice when compared to B6 mice. However, similar to results seen during coinfection with Spn IT + Lm PO, co-treatment in *Ncr1*^*iCre*^x*Il10*^fl/fl^ mice with HK Spn or LPS did not restore Lm burdens to the level seen in Lm PO only infected mice, suggesting there may be other mechanisms that contribute to peripheral burdens of Lm during co-treatment. Overall, these data suggest that some, but not all, pulmonary stimuli can increase susceptibility to oral Lm in a manner that is partially dependent on the production of IL-10 by *Ncr1*+ cells.

## Discussion

Previous efforts to model oral Lm infection in mice have shown that despite high inoculation doses, the spread of Lm to peripheral tissues was limited [20]. Likewise, most humans appear to be highly resistant to oral infection by this pathogen [15]. It has thus been unclear what factors might render humans or animals susceptible to developing more severe listeriosis. Our data here indicate that respiratory coinfection or lung exposure to agents that trigger regulatory NK cell responses dramatically increase susceptibility of mice to oral infection by Lm. Of note is that the site of secondary inoculation is especially susceptible to Lm replication, as seen by an increase in peripheral Lm burdens in the lungs of coinfected or co-treated mice when compared to oral Lm infection alone (Figs 2F and 6E and S12C and S12F). In addition, the production of IL-10 by *Ncr1*^+^ cells was shown to be essential for the increase in host susceptibility and was associated with the suppression of both myeloid cell accumulation and their phagocytic capacity in Lm-infected tissues. These results support the model that microbes or other environmental factors which promote innate lymphoid cell production of IL-10 can actively increase host susceptibility to intestinal infection by Lm, and likely other pathogens.

Using a mixture of genetically tagged Lm strains, it has been estimated that 1 per million Lm adhere to and cross the gut during oral infection [42,43]. Based on this frequency, the oral infection dose of 5x10^8^ used here likely resulted in only ~500 Lm accessing the periphery. While there may be some expansion of these bacteria after they cross the intestinal barrier, the peripheral immune response in most cases appeared to be highly effective at limiting Lm expansion and ultimately resolving the infection with minimal effects on morbidity (Fig 1A). The efficient control of Lm burdens in our model of oral Lm infection was presumably dependent on an influx of inflammatory monocytes and neutrophils (as seen in Fig 5), which are known to be important in the early management and clearance of invasive Lm [44,45]. In addition, the rapid containment of Lm by the systemic immune response prohibited the accumulation of systemic cytokines (S2 Fig) and thus prevented host malaise. While others have seen an increase in susceptibility to oral Lm infection through the use of a murinized strain of InlA Lm, here we show that this strain did not enhance the spread of InlA^m^ to peripheral tissues (Fig 1). This may be due to the strain of mice as well as the specific infection protocol used by others, including CaCO_3_ treatment to impact stomach pH, or extending fasting of the mice before infection. In addition, although the 10403S strain of Lm used in these studies was derived from a clinical isolate, it is also worth noting that strain variability may impact the progression of disease in both mice and humans. Here we propose that efficient bacterial clearance and an absence of a systemic cytokine response explains why most humans who consume Lm-infected foods fail to develop symptoms, resulting in the disconnect between the low frequency of clinical listeriosis and the much higher frequency of Lm consumption and shedding by healthy humans [15].

Human populations with heightened incidence of clinical listeriosis include the elderly, pregnant women, and immunocompromised individuals–including cancer patients [10,23]. However, severe listeriosis does occur in individuals outside of these criteria. This suggests that there are additional predisposing factors that dictate the susceptibility of a given host to Lm infection. Our data here suggest that exposure to pulmonary stimuli, either Spn or L1S, that trigger the production of IL-10 by *Ncr1*+ cells could be one such factor that increases the survival of invasive Lm (Figs 3, 4 and 6). Interestingly, while Spn and L1S exposure broaden the list of known factors that impact susceptibility to oral Lm infection, the mechanisms by which they increase susceptibility may be conserved. Observational studies in humans as well as reports in animal models have separately shown that age, pregnancy, and cancer were associated with changes in IL-10 and in myeloid cells. For example, serum IL-10 and IL-10-producing Tfh cells have been shown to accumulate with age [46]. NK cell populations have also been shown to be skewed in aged individuals, with reductions in their cytotoxicity and an increase in the accumulation of the CD56dim subset [47,48]. Elderly populations are also at higher risk for severe Spn infections and are frequently colonized with this bacterium [49]. Findings from our studies suggest that the subset of elderly individuals who develop severe Lm infections may be co-colonized with Spn, have heightened basal levels of serum IL-10, or altered NK cell function that predisposes NK cells to produce more IL-10. Pregnancy in both humans and mice is also known to skew immune homeostasis toward an anti-inflammatory environment [50]. Successful pregnancy in mice and humans is integrally tied to the production of IL-10 by NK cells, where spontaneous abortions are reported when uterine NK cells are disrupted in their ability to produce IL-10 [51,52,53]. Consistent with pregnancy reducing resistance of C57BL/6J mice to oral Lm infection [54], Abram et al. showed that Lm infection during pregnancy significantly increased serum IL-10 when compared to virgin mice [55]. Finally, in regard to cancer patients, Becattini et al. have shown that the combination of chemotherapeutic drugs in mice skewed the gut microbiota and markedly decreased the number of circulating immune cells, including inflammatory monocytes, both of which may contribute to heightened susceptibility to oral Lm infection [21]. It will be important to further investigate the extent to which susceptible populations are predisposed to IL-10 production, or may also be exposed to Spn or other microbial or environmental agents that specifically enhance the production of IL-10 by NK cells.

Coinfections have been shown to worsen host outcomes in a variety of disease settings. In a recent meta-analysis review, Langford et al. report secondary bacterial infections in admitted patients that ranged from 5.9% of all hospitalized COVID-19 patients, to 8.1% of critically ill patients [56]. Similarly, in the 2009 H1N1 influenza pandemic, 12% of hospitalized patients were found to carry a secondary infection, and this number increased to 30% in critically ill patients [57,58]. An increase in disease severity in coinfected patients can be attributed to alterations of the immune response. For example, coinfection with H1N1 influenza and dengue virus in mice resulted in increased pathophysiology, differential chemokine expression, and reduced monocyte recruitment to the lungs [59]. In humans with latent Mtb and helminth infection, Rajamanickam et al. report an overall impairment in monocyte function, with a decrease in phagocytic activity and a shift towards an M2 phenotype, and diminished pro-inflammatory cytokine production but with an increase in IL-10 production. Interestingly, in this study, when anthelmintic therapy was introduced, a restoration in monocyte activity and pro-inflammatory cytokine production was observed [60]. Thus, coinfections have often been tied to alterations in the immune response through the dampening of myeloid cell activity and the induction of anti-inflammatory responses. Our data here are consistent with this theme, where coinfection with Spn and Lm limits myeloid cell accumulation as well as myeloid cell phagocytic capacity in infected tissues. It will thus be of interest to discern whether NK cell IL-10 production plays a role in other coinfection models by impacting myeloid cell function in a manner that increases disease severity.

Many infections that involve pathogens that infect the lungs have also been shown to cause perturbations in the gut. In some cases, these effects may be due to the replication of respiratory pathogens in the intestines, or due to the lung pathogen altering gut integrity or the gut microbiome. For example, clinical case reports have noted that coronaviruses, including COVID-19, can cause gastrointestinal symptoms in up to 50% of infected individuals [1]. Of note is that SARS-CoV-2 enters cells through binding the angiotensin I converting enzyme 2 receptor (ACE2r) on host cells, which is highly expressed by enterocytes of the small intestines [61]. Additionally, viral shedding has also been detected in the feces of infected individuals [62]. Influenza infection has similarly been associated with gastrointestinal symptoms [63]. In mouse models, influenza infection also caused intestinal injury through microbiota-dependent inflammation [64]. While not known to replicate in the intestines, high doses of Spn (2x10^8^) have been shown to cause transient increases in gut permeability [65]. In our study the lower dose of Spn used in our experiments did not impact gut barrier permeability when compared to Lm PO infection alone (Fig 2G). However changes in permeability could promote the dissemination of Lm or other intestinal pathogens in specific individuals or contexts, and additional investigation is needed to more fully address what impact pulmonary infections could have on the gut. Of further interest is the fact that localized pulmonary stimuli (including L1S, HK Spn, and LPS) that lack the ability to replicate can increase susceptibility to oral Lm infection (Figs 6 and S12). Whether these stimuli impact oral Lm infection at the level of the gut is not known. Nevertheless, additional work is needed to fully define the variety of mechanisms by which respiratory pathogens or stimuli act to alter susceptibility to oral infections by intestinal pathogens.

Both genetic and environmental factors influence the overall susceptibility of an individual host to infection by microbial pathogens. Thus, while a given pathogen may cause severe disease in one individual, the same pathogen may fail to elicit symptoms in another. Our work here has highlighted the potential for pulmonary coinfection or immune stimulation to exacerbate susceptibility to infection by a pathogen that enters the body through the intestinal tract. In this context, NK cells are the predominant producers of IL-10 in response to Spn infection, and this IL-10 acts to skew myeloid cell responses and phagocytic capacity to facilitate greater survival and replication of systemic Lm. NK cell production of IL-10 is now known to be elicited by systemic infection by a variety of infectious agents, including mouse cytomegalovirus, *Leishmania donovanii*, *Toxoplasma gondii*, and *Yersinia pestis* [66,67,68,69]. Currently, the mechanisms that govern this IL-10 production are not well defined. However, efforts to elucidate such mechanisms could lead to development of therapies that bolster resistance of susceptible populations to Lm and other mucosal pathogens that exploit regulatory NK cell activity.

## Materials and methods

### Ethics statement

All animal procedures were approved by the University of Colorado Anschutz Medical Campus’s Animal Care and Use Committee (Approval #: 313). CO_2_ asphyxiation or pentobarbital were used for animal sacrifice. These protocols adhere to standards of the United States Public Health Service and Department of Agriculture.

### Mice

C57BL/6J (B6) mice (male and female, 8–12 weeks old) were either directly purchased from Jackson Laboratory or were bred and maintained in the University of Colorado specific pathogen free (SPF) animal facilities. B6.*tiger* (*IL10*-gfp reporter) mice (male and female, 8–12 weeks old) were purchased from Jackson Laboratory and bred and maintained in the University of Colorado SPF animal facilities. *Ncr1*^*iCre*^x*Il10*^fl/fl^ mice (male and female, 8–12 weeks old), where mice with floxed *Il10* were crossed to a strain expressing an improved Cre (iCre) driven by the Ncr1 gene (*Ncr1*^*iCre*^) [70,71], were a generous gift from Dr. Joseph Sun (Sloan Kettering Institute, NY). Mice were bred and maintained in the University of Colorado SPF animal facilities.

### Bacterial strains and growth conditions

Frozen stocks of WT *Listeria monocytogenes* (Lm) strain 10403S (serotype 1/2a), murinized internalin A Lm (InlA^m^ Lm) on a 10403S WT background, or congenic p60-deficient Lm (Δp60 Lm) on the 10403S WT background were thawed, diluted in tryptic soy broth (TSB) (MP Biomedicals) supplemented with 100 μg/mL streptomycin (Sigma), and grown overnight at 37°C in a shaker set to 220 rpm. The next morning, Lm was back diluted and grown to log phase for infections. Frozen stocks of *Streptococcus pneumoniae* (Spn), Type 2 strain D39, which were a kind gift from Dr. Edward Janoff (University of Colorado School of Medicine), were thawed from frozen stocks, diluted in Todd-Hewitt broth (RPI Research Products) plus 0.5% yeast extract (BD Biosciences), and grown to mid-log phase without shaking in a 37°C, 7.5% CO2 incubator.

### Preparation of L1S protein

L1S protein was expressed in an E. coli construct with a pTrcHis (Invitrogen) and purified. Briefly, log phase bacteria were incubated for 8 hours with IPTG (Life Technologies) to induce protein expression. Following cell lysis and DNAse treatment, lysates were centrifuged to pellet cellular debris. Supernatants were filtered through a 0.22 μm syringe filter (Fisher Scientific) and loaded onto an ATKA FPLC for nickel (Ni) column (GE Healthcare) for affinity purification of the His-tagged protein. Fractions from the eluted column were evaluated using SDS-PAGE and Coomassie staining. Fractions containing the L1S protein were concentrated using Millipore 10k KD columns (Millipore) and protein concentrations were determined using a BCA assay (Thermo Scientific).

### Infections and treatments

For intravenous Lm infections, log phase bacteria were diluted in sterile PBS, and 5x10^3^ colony-forming units (CFUs) Lm in 200ul final volume were injected intravenously (IV) into the lateral tail vein. For oral gavage Lm infections, log phase bacteria were diluted in sterile PBS, and 5x10^8^ CFUs Lm in 200ul final volume were gavaged intragastrically (PO) using an oral gavage needle (20 gauge x 1.5”, Fisherbrand) into mice anaesthetized using an isoflurane anesthesia vaporizer (VetFlo) and induction box. For intratracheal Spn infections, log phase bacteria were diluted in PBS, and 10^5^ CFUs Spn in 50ul final volume were intratracheally instilled (IT) into the lungs using a blunted needle in mice that were anaesthetized using an isoflurane anesthesia vaporizer and induction box.

For intratracheal L1S instillation, protein was diluted into sterile PBS to a final concentration of 100ug/50ul and IT instilled into the lungs using a blunted needle in mice that were anaesthetized using an isoflurane anesthesia vaporizer and induction box. To heat kill Spn (HK Spn), 2x10^7^ Spn/ml were resuspended in sterile PBS, and heat-killed in a 56°C water bath for 1 hour. Heat inactivation was confirmed by plating 100ul HK Spn overnight on TSB agar plates supplemented with neomycin (10 μg/mL) and catalase (5,000 Units per plate) (Worthington) and grown overnight in a 7.5% CO2 incubator at 37°C, to ensure no colonies formed. For intratracheal instillation of HK Spn, 10^6^ HK Spn/50ul was IT instilled into the lungs using a blunted needle in mice that were anaesthetized using an isoflurane anesthesia vaporizer and induction box. Additional intratracheal treatments included lipopolysaccharide (LPS, List Labs), polyinosinic:polycytidylic acid (poly:IC, Enzo) and Pam3CysSerLys4 (Pam3CSK4, invivoGen). In each case, the reagent was diluted into sterile PBS to a final concentration of 50ug/50ul and IT instilled into the lungs using a blunted needle in mice that were anaesthetized using an isoflurane anesthesia vaporizer and induction box.

For coinfection experiments, anaesthetized mice were first IT instilled with 50ul Spn, L1S, HK Spn, LPS, polyI:C, or Pam3CSK4 followed by intragastric gavage with 200ul Lm. This protocol was optimized using Trypan Blue (MP Biomedicals) and confirmed minimal inhalation of Lm into the lungs. For mice infected with Lm only, mice were first IT instilled with 50ul PBS followed by PO gavage with 200ul Lm, whereas for mice infected with Spn only, or L1S only, mice were IT instilled with 50ul Spn or 50ul L1S followed by PO gavage with 200ul PBS.

### Enumeration of CFUs in mouse organs

For CFU enumeration from spleens, livers, and lungs, tissues were aseptically harvested into PBS, weighed for total weight, partitioned, and weighed again for later CFU calculations. A portion of each tissue was then placed into 0.02% Nonidet P-40 (NP-40) (Sigma) for Lm CFU enumeration, while the other half was placed into PBS for Spn CFU enumeration. For both bacteria, organs were homogenized for 45 seconds with a tissue homogenizer (IKA Works). Between groups, homogenization blades were sterilized with 70% ethanol for 30 seconds and rinsed with water for 15 seconds. For Lm CFU enumeration, serial dilutions were plated on TSB agar plates supplemented with streptomycin (100 μg/mL) and grown overnight at 37°C. For Spn CFU enumeration, serial dilutions were plated on TSB agar plates supplemented with neomycin (10 μg/mL) and catalase (5,000 Units per plate) (Worthington) and grown overnight in a 7.5% CO2 incubator at 37°C. Total CFUs were calculated using the following formula: total CFU per organ = CFU x (total tissue weight/weight used). For Lm CFU enumeration from mesenteric lymph nodes (MLNs), 3 MLNs were aseptically harvested into 1 ml NP-40 with 1mm metal beads (Next Advance) and homogenized for 4 mins using a bullet homogenizer (Next Advance). Serial dilutions were plated on TSB agar plates supplemented with streptomycin (100 μg/mL) and grown overnight at 37°C.

For Lm CFU enumeration from intestines, entire intestines were aseptically harvested into sterile PBS. On a dry petri dish, forceps were used to squeeze out colon contents, and colon contents were transferred into pre-weighed tubes containing 1ml NP-40 with 1mm metal beads (Next Advance). Tubes were weighed again to calculate total weight of colon contents, then homogenized for 4 mins using a bullet homogenizer (Next Advance). The remaining intestines were then cut into ileum and colon sections and separated for further processing. Each portion was flushed with sterile PBS using a 25-gauge needle, then cut transversely to open intestines, rinsed several times with sterile PBS, then minced to approximately 3-5mm sized pieces. Ileal or colon tissues were then transferred into tubes containing 1ml NP-40 with 1mm metal beads (Next Advance), then homogenized for 4 mins using a bullet homogenizer (Next Advance). Serial dilutions were plated on TSB agar plates supplemented with streptomycin (100 μg/mL) and grown overnight at 37°C.

### FITC-dextran treatment

FITC-labeled dextran (4kDa; Sigma-Aldrich) was used to assess in vivo intestinal permeability in sham infected, Lm PO infected, or coinfected mice. 4 hr prior to harvest, mice were deprived of food and water, and gavaged using an oral gavage needle (20-gauge x 1.5”, Fisherbrand) with 100ul of 200mg/ml FITC-dextran. Upon sacrifice, blood was collected by cardiac puncture into polypropylene tubes (Sarstedt), spun at full speed for 10 mins, and serum was used to measure fluorescence intensity. Fluorescence was measured using an excitation wavelength of 493nm and an emission wavelength of 518 nm.

### Serum cytokine production

Upon sacrifice, blood was collected by cardiac puncture into polypropylene tubes (Sarstedt) and allowed to clot at room temperature. Tubes were centrifuged at full speed, and serum was collected and frozen at -20°C prior to analysis. Cytokine levels were assayed using a 10-plex Pro-inflammatory Cytokine kit (Meso Scale Diagnostics). Assays were performed per manufacturer’s instructions, read using a 1300 MESO QuickPlex SQ 120 (Meso Scale Diagnostics), and analyzed using Discovery Workbench (Meso Scale Diagnostics). Commercially available ELISA kits for CCL2 and CCL3 (LifeTech) were used to measure serum CCL2 and CCL3 protein levels. A commercial ELISA kit for IL-10 (BD Biosciences) was used to measure serum IL-10 protein levels.

### Single cell isolation

Upon sacrifice, spleens and livers were aseptically harvested into PBS. Mice were then perfused with 10ml sterile PBS before removal of lungs. All tissues were weighed for total weight, partitioned and weighed again for later total cell number calculations. Spleens, livers, and lungs were finely chopped in 12-well plates (VWR) and 3 ml collagenase in HBSS+ (Life Technologies) was added to each tissue sample. For spleens and livers, type IV collagenase (1mg/ml, Worthington) was used, while for lungs, type VIII collagenase (1mg/ml, Sigma-Aldrich) was used. After 25 mins incubation at 37°C, 60ul 0.5mM EDTA was added to each sample to stop the digestion. Using HBSS- media, spleens were passaged through a 70um cell strainer, while livers and lungs were passaged through a 100um cell strainer. Spleen and lung cell suspensions were treated with 3ml RBC lysis buffer for 5 mins, quenched with 10ml HBSS-, centrifuged at 500g for 5 mins, resuspended into 2ml HBSS-without cations, counted for total cell number calculations, and 2x10^6^ cells were plated and used for FACS analysis, see below. Following passage through a 100um cell strainer, liver cells were resuspended in 40% Percoll (Fisher Scientific) diluted in HBSS- media. The 40% Percoll was then underlayed with 60% Percoll and centrifuged for 20 mins at room temperature at 1300g with the lowest settings for acceleration and deceleration. After centrifugation, cells at the layer interface were collected and washed, treated with 3ml RBC lysis buffer for 5 mins, quenched with 10ml HBSS-, centrifuged at 500g for 5 mins, resuspended into 200ul HBSS-, counted for total cell number calculations, and 2x10^6^ cells were plated and used for FACS analysis.

### Flow cytometry

For analysis of IL-10-gfp^+^ staining in B6.*tiger* (*IL10*-gfp reporter) mice, single cell suspensions were incubated for 3 hours in RP10 media (RPMI 1640, 10% FBS, 1% L-glutamine, 1% Sodium Pyruvate, 1% Penicillin, 1% Streptomycin and 0.1% β-ME) containing Brefeldin A (GolgiPlug, BD Biosciences). No additional *ex vivo* stimulation was used for NK cell analysis. After the 3 hour Brefeldin A treatment, cells were washed with PBS, and stained for 30 mins with a live/dead stain (1:500, BV510, LIVE/DEAD Fixable Aqua Dead Cell Stain, Invitrogen), washed again with PBS, and prepared for blocking. Cells were incubated in anti-CD16/32 (2.4G2 hybridoma supernatant) for 20 mins to block Fc receptors, then stained for 30 mins with extracellular fluorophore-labeled antibodies diluted in FACS buffer (1% BSA and 0.01% NaN_3_ in PBS). Extracellular staining antibodies included: anti-CD45.2 (1:100, clone 104, BUV395, BD Biosciences), B220 (1:300, clone RA3-6B2, APCCy7, ebioscience), CD3 (1:300, clone 17A2, PE, Biolegend), and NK1.1 (1:200, clone PK136, APC, ebioscience). After extracellular staining, cells were fixed and permeabilized in 1% paraformaldehyde supplemented with Saponin for 20 mins. To amplify the IL-10gfp signal, cells were next stained with a rabbit monoclonal anti-GFP antibody (1:100, Invitrogen), followed by a goat anti-rabbit IgG Alexa Fluor 488 antibody (1:50, Invitrogen). For analysis of myeloid cells, single cell suspensions were blocked in anti-CD16/32 (2.4G2 hybridoma supernatant) for 20 mins, then stained for 30 mins with extracellular fluorophore-labeled antibodies diluted in FACS buffer (1% BSA and 0.01% NaN_3_ in PBS). Extracellular staining antibodies included: anti-CD45.2 (1:100, clone 104, BUV395, BD Biosciences), CD11b (1:200, clone M1/70, APCCy7, Invitrogen), Ly6C (1:200, clone HK1.4, BV421, BioLegend), CD11c (1:200, clone N418, PerCP5.5, BioLegend), and Ly6G (1:200, clone 1A8, PE, BioLegend). For analysis of bioparticle uptake and phagosomal maturation in myeloid cells, single cell suspensions were plated at 2x10^6^ cells/well in 150ul RPMI. pHrodo *Green S*. *aureus Bioparticles* (ThermoFisher) were reconstituted as specified by manufacturer’s protocol to a concentration of 1mg/ml, and 50ul were added per well. Cells were then incubated for 1 hour at 37°C. After 1 hour, cells were washed with ice-cold FACS buffer to stop phagocytosis, and kept on ice through the Fc block and extracellular staining steps (as specified above). At least 100,000 events per sample were collected using a LSR Fortessa (BD Biosciences). Flow data were processed using FlowJo software (TreeStar).

### Quantification and statistical methods

Graphing and statistical analyses were performed using Prism (GraphPad Software). For mouse microbial CFUs, statistical significance was assessed by Mann-Whitney test. In other experiments that compared between more than two datasets, a one-way or a two-way ANOVA analysis of variance with Tukey’s multiple-comparison test were performed. All data are representative of 2–3 independent experiments, as specified in the figure legends, with the specified number of mice per group. p<0.05 was considered significant. Data for all experiments are presented as the mean ± standard deviation (SD) or mean ± standard deviation (SEM).

## Supporting information

S1 Fig Trypan Blue oral and intratracheal inoculation. C57BL/6J (B6) mice were treated in panel 1: 200ul Trypan Blue PO, panel 2: 50ul Trypan blue IT, panel 3: 50ul PBS IT followed immediately by 200ul Trypan Blue PO. Mice were sacrificed 10 mins after treatment, and tissues were collected and photographed.
(TIF)

S2 Fig Mesoscale and ELISA measurement of cytokines/chemokines from Day 3 serum. (A-J) Serum from C57BL/6J (B6) male and female age-matched mice that were infected with sham control, Lm PO, Spn IT, or Spn IT + Lm PO were measured by Mesoscale for 10 cytokines. Data represent mean ± SD, One-way ANOVA, pooled from 2 experiments, 4–6 mice per group, *p<0.05, **p<0.002, ***p<0.0002 between indicated groups. (K-L) Serum from C57BL/6J (B6) male and female age-matched mice that were infected with sham control, Lm PO, Spn IT, or Spn IT + Lm PO were measured by commercial ELISA kits for protein concentrations of CCL2 and CCL3. Data represent mean ± SD, One-way ANOVA, pooled from 2 experiments, 6–8 mice per group, *p<0.05, ****p<0.0001 between indicated groups.
(TIF)

S3 Fig Flow cytometry plots for the detection of lymphocytes. Representative flow cytometry plots from the spleen of a B6.*tiger* (*Il10*-gfp reporter) mouse infected with Lm PO and harvested at 3 dpi.
(TIF)

S4 Fig Flow cytometry plots for detection of Il10-gfp reporter staining in lymphocytes. Representative flow cytometry plots from the spleens of B6.*tiger* (*Il10*-gfp reporter) mice infected with sham control, Lm PO, Spn IT, or Spn IT + Lm PO and harvested at 3 dpi.
(TIF)

S5 Fig Flow cytometry plots for the detection of inflammatory monocytes and neutrophils. Representative flow cytometry plots from the spleen of a B6.*tiger* (*Il10*-gfp reporter) mouse infected with Lm PO and harvested at 3 dpi.
(TIF)

S6 Fig Flow cytometry plots for detection of Il10-gfp reporter staining in inflammatory monocytes and neutrophils. Representative flow cytometry plots from the spleens of sham infected control B6 mice and B6.*tiger* (*Il10*-gfp reporter) mice infected with sham control, Lm PO, or Spn IT + Lm PO and harvested at 3 dpi.
(TIF)

S7 Fig Lm bacterial burdens and IL-10-gfp reporter staining in male versus female age-matched tiger mice. B6.*tiger* (*Il10*-gfp reporter) male and female age-matched mice were infected with sham control, Lm PO, Spn IT, or Spn IT + Lm PO and harvested at 3 dpi. Lm burdens were enumerated from (A) spleens, (B) livers, and (C) lungs. Data represent mean ± SD, Mann-Whitney t-test, pooled from 2 experiments, 3–4 mice per group. (D) Quantitation of percent CD3^-^NK1.1^+^ NK cells that stained positive for IL-10-gfp^+^ in spleens, livers, and lungs. Data represent mean ± SD, Two-way ANOVA, pooled from 2 experiments, 3–4 mice per group.
(TIF)

S8 Fig Spn burdens during coinfection with WT Lm PO versus Δp60 Lm PO. Spn burdens from mice in Fig 3E, 3F, and 3G were enumerated in (A) spleens, (B) livers, (C) lungs. Data represent mean ± SD, Mann-Whitney t-test, pooled from 2 experiments, 4 mice per group.
(TIF)

S9 Fig Leukocyte analysis from livers of B6 and Ncr1^iCre^xIl10^fl/fl^ infected mice. C57BL/6J (B6) and *Ncr1*^*iCre*^x*Il10*^fl/fl^ male and female age-matched mice were infected with sham control, Lm PO, or Spn IT + Lm PO and livers were harvested at 3 dpi. (A) Quantitation of percent CD45.2^+^Ly6C^+^CD11b^+^ leukocytes. Data represent mean ± SD, One-way ANOVA, pooled from 3 experiments, 6–12 mice per group. Representative flow plots of Ly6G^+^ neutrophils and Ly6G^-^CD11c^l^° inflammatory monocytes in B6 and *Ncr1*^*iCre*^x*Il10*^fl/fl^ coinfected mice. Quantitation of total number of CD45.2^+^Ly6C^+^CD11b^+^ cells that are Ly6G^-^CD11c^l^° inflammatory monocytes (C) or Ly6G^+^ neutrophils (D). Data represent mean ± SD, One-way ANOVA, pooled from 3 experiments, 6–12 mice per group, *p<0.05 between indicated groups.
(TIF)

S10 Fig Percent pHrodo positive inflammatory monocytes and neutrophils with representative flow cytometry plots of pHrodo expression. pHrodo *S*. *aureus* Bioparticles were incubated for 1 hr with cells isolated from spleens and lungs. Percent pHrodo positive inflammatory monocytes and neutrophils were determined by flow cytometry. %pHrodo+ from inflammatory monocytes in spleens (A), neutrophils in spleens (C), inflammatory monocytes in lungs (E), and neutrophils in lungs (G). Data represent mean ± SD, One-way ANOVA, pooled from 2 experiments, 8–9 mice per group, *p<0.05 between indicated groups. Representative pHrodo staining from inflammatory monocytes in spleens (B), neutrophils in spleens (D), inflammatory monocytes in lungs (F), and neutrophils in lungs (H). In panel 1: Light gray shaded line represents Sham infected B6 mice with no pHrodo staining, Black line represents Sham infected B6 mice with pHrodo staining, Blue line represents Lm PO infected B6 mice with pHrodo staining, Pink line represents Spn IT + Lm PO infected B6 mice with pHrodo staining. In panel 2: Pink shaded line represents Spn IT + Lm PO infected B6 mice with pHrodo staining, while Pink dotted line represents Spn IT + Lm PO infected *Ncr1*^*iCre*^x*Il10*^fl/fl^ mice with pHrodo staining.
(TIF)

S11 Fig Flow cytometry plots for detection of Il10-gfp reporter staining in lymphocytes. Representative flow cytometry plots from the spleens of B6.*tiger* (*Il10*-gfp reporter) mice infected with sham control, Lm PO, L1S IT, or L1S IT + Lm PO and harvested at 3 dpi.
(TIF)

S12 Fig Peripheral Lm burdens upon co-treatment with alternative pulmonary stimuli. C57BL/6J (B6) male and female age-matched mice were infected with Lm PO or 10^6^ HK Spn IT + Lm PO, and CFUs were determined 3 dpi. Lm burdens were enumerated from (A) spleens, (B) livers, and (C) lungs. Data are represented as mean ± SD, Mann-Whitney t-test, pooled from 3 experiments, with 10–12 mice per group, ****p<0.0001 between indicated groups. B6 male and female age-matched mice were infected with Lm PO, or co-treated with 50ug LPS IT + Lm PO, 50ug poly:IC IT + Lm PO, or 50ug Pam3CSK4 IT + Lm PO and CFUs were determined 3 dpi. Lm burdens were enumerated from (D) spleens, (E) livers, and (F) lungs. Data are represented as mean ± SD, Mann-Whitney t-test, pooled from 2 experiments, with 8–10 mice per group, **p<0.002, ***p<0.0002, ****p<0.0001 between indicated groups. C57BL/6J (B6) and *Ncr1*^*iCre*^x*Il10*^fl/fl^ male and female age-matched mice were infected with Lm PO, 10^6^ HK Spn IT + Lm PO, or 50ug LPS IT + Lm PO and harvested at 3 dpi. Lm burdens were enumerated from (G) spleens, (H) livers, and (I) lungs. Data represent mean ± SD, Mann-Whitney t-test, pooled from 2 experiments, 4–8 mice per group. **p<0.002, ***p<0.0002, between indicated groups, #p<0.05, ##p<0.001 between indicated co-treated *Ncr1*^*iCre*^x*Il10*^fl/f^ mice and B6 Lm PO infected mice.
(TIF)
