# Supplementary figures and images for: Pulmonary insults exacerbate susceptibility to oral Listeria monocytogenes infection through the production of IL-10 by NK cells
Source: PLoS Pathog. 2021 Apr 20;17(4):e1009531. doi: 10.1371/journal.ppat.1009531 (PMC8087096; doi:10.1371/journal.ppat.1009531)

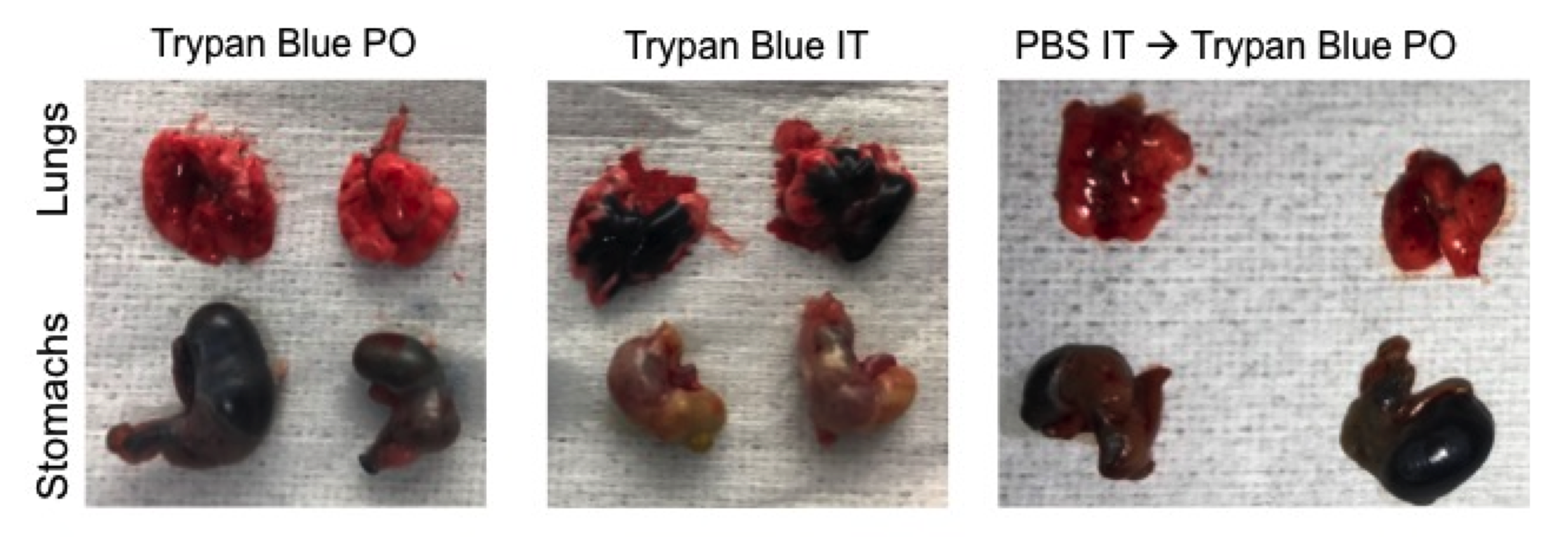

Supplement: S1 Fig — C57BL/6J (B6) mice were treated in panel 1: 200ul Trypan Blue PO, panel 2: 50ul Trypan blue IT, panel 3: 50ul PBS IT followed immediately by 200ul Trypan Blue PO. Mice were sacrificed 10 mins after treatment, and tissues were collected and photographed. (TIF) [file ppat.1009531.s001.tif]

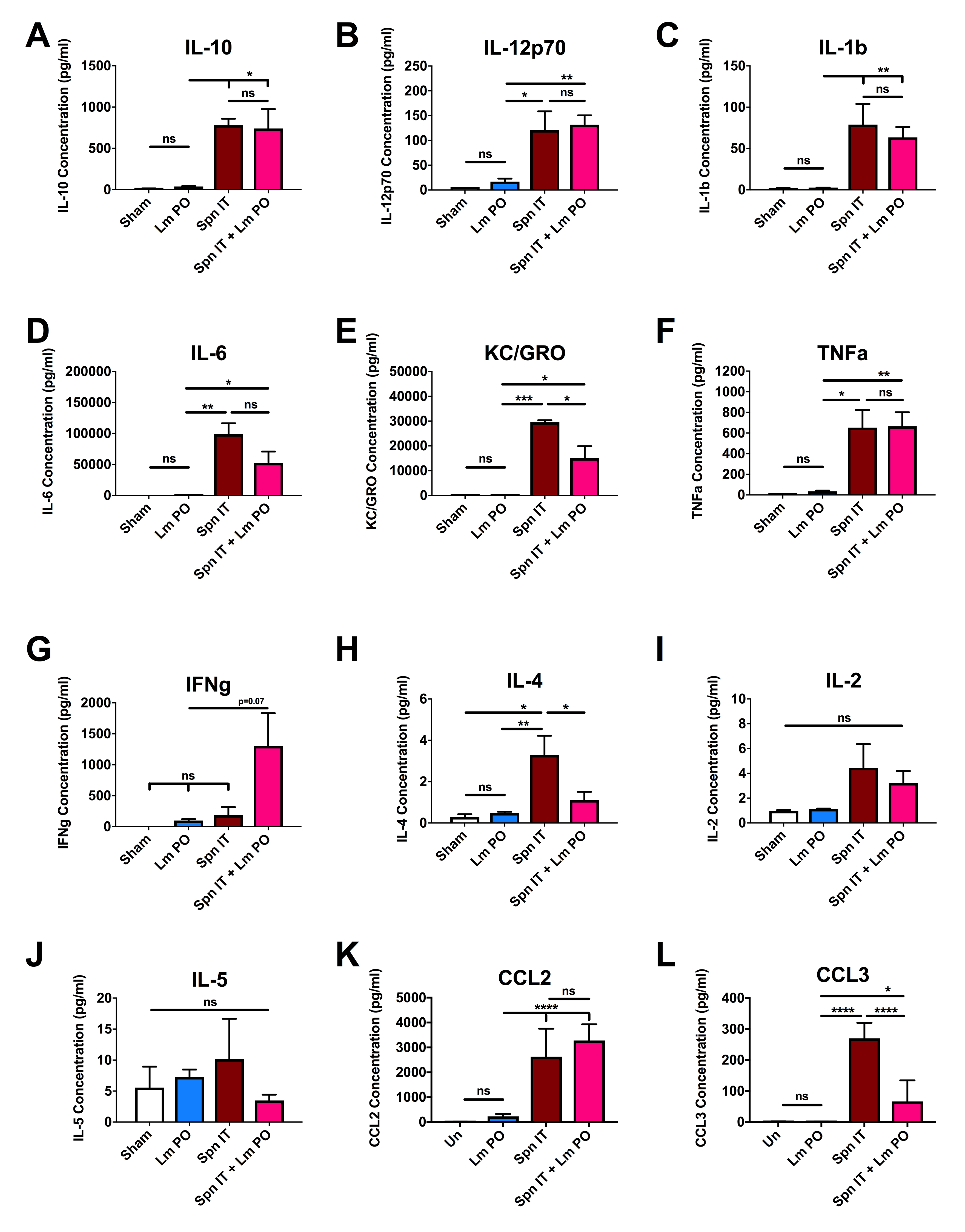

Supplement: S2 Fig — (A-J) Serum from C57BL/6J (B6) male and female age-matched mice that were infected with sham control, Lm PO, Spn IT, or Spn IT + Lm PO were measured by Mesoscale for 10 cytokines. Data represent mean ± SD, One-way ANOVA, pooled from 2 experiments, 4–6 mice per group, *p<0.05, **p<0.002, ***p<0.0002 between indicated groups. (K-L) Serum from C57BL/6J (B6) male and female age-matched mice that were infected with sham control, Lm PO, Spn IT, or Spn IT + Lm PO were measured by commercial ELISA kits for protein concentrations of CCL2 and CCL3. Data represent mean ± SD, One-way ANOVA, pooled from 2 experiments, 6–8 mice per group, *p<0.05, ****p<0.0001 between indicated groups. (TIF) [file ppat.1009531.s002.tif]

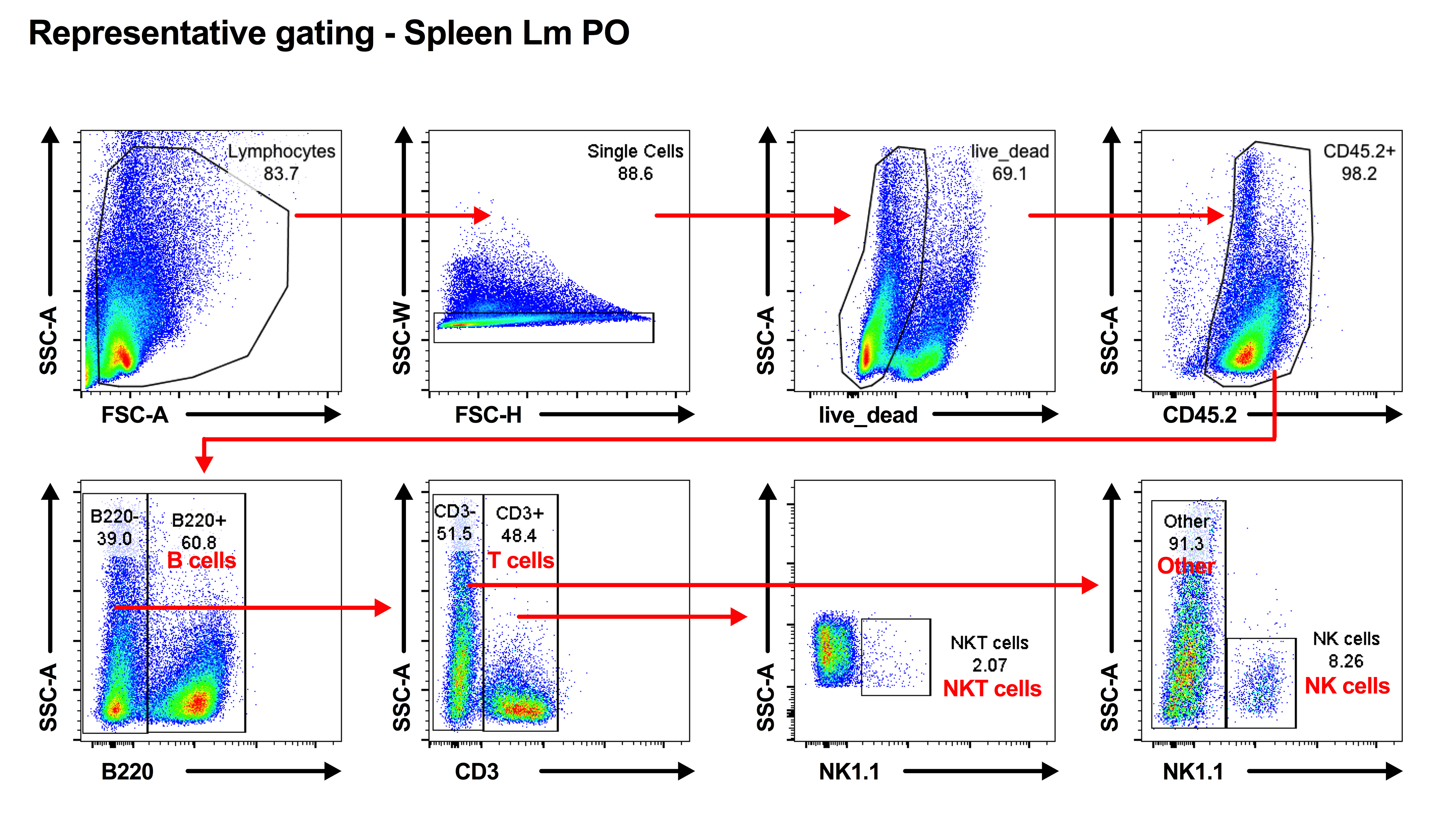

Supplement: S3 Fig — Representative flow cytometry plots from the spleen of a B6.tiger (Il10-gfp reporter) mouse infected with Lm PO and harvested at 3 dpi. (TIF) [file ppat.1009531.s003.tif]

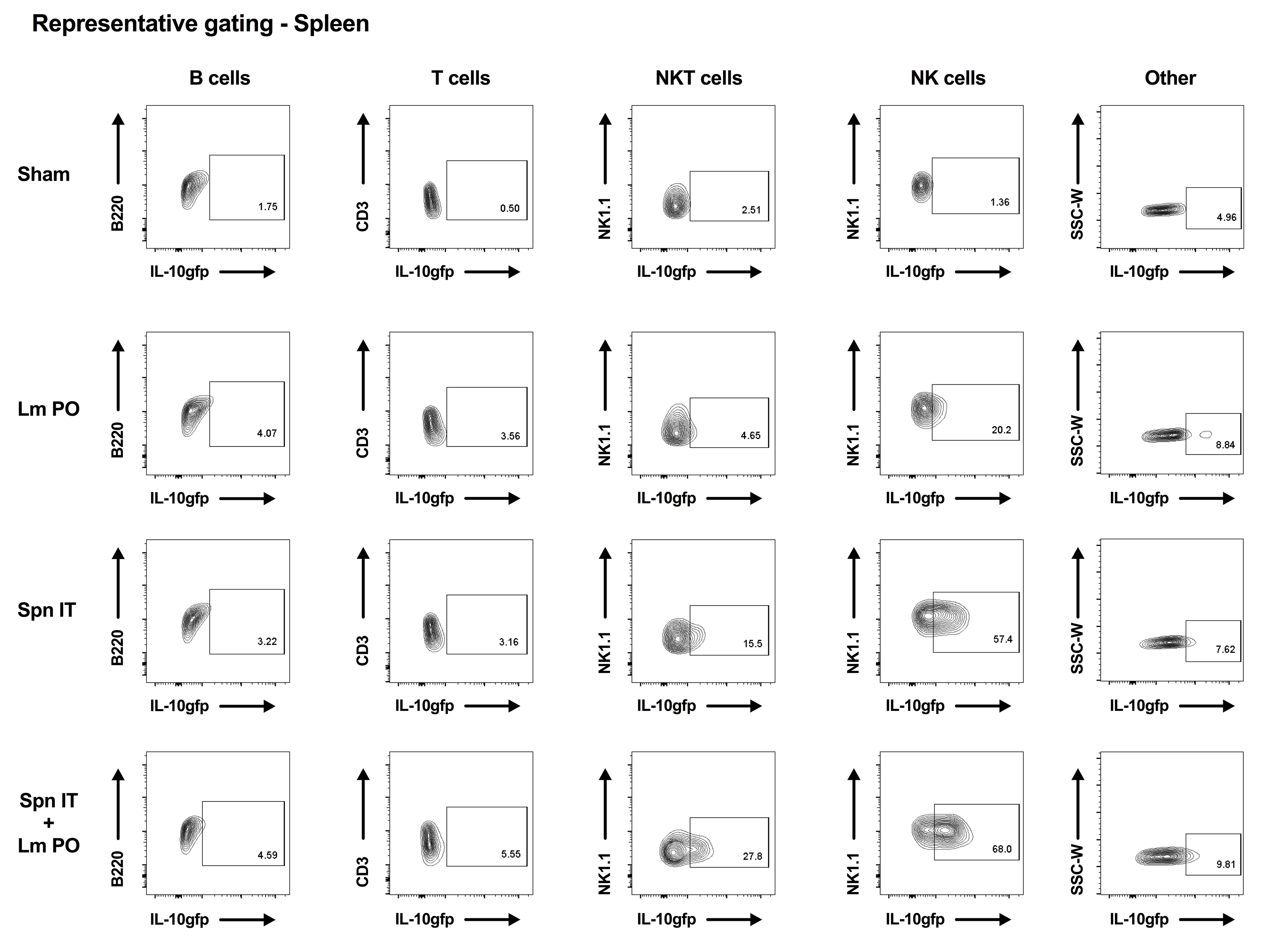

Supplement: S4 Fig — Representative flow cytometry plots from the spleens of B6.tiger (Il10-gfp reporter) mice infected with sham control, Lm PO, Spn IT, or Spn IT + Lm PO and harvested at 3 dpi. (TIF) [file ppat.1009531.s004.tif]

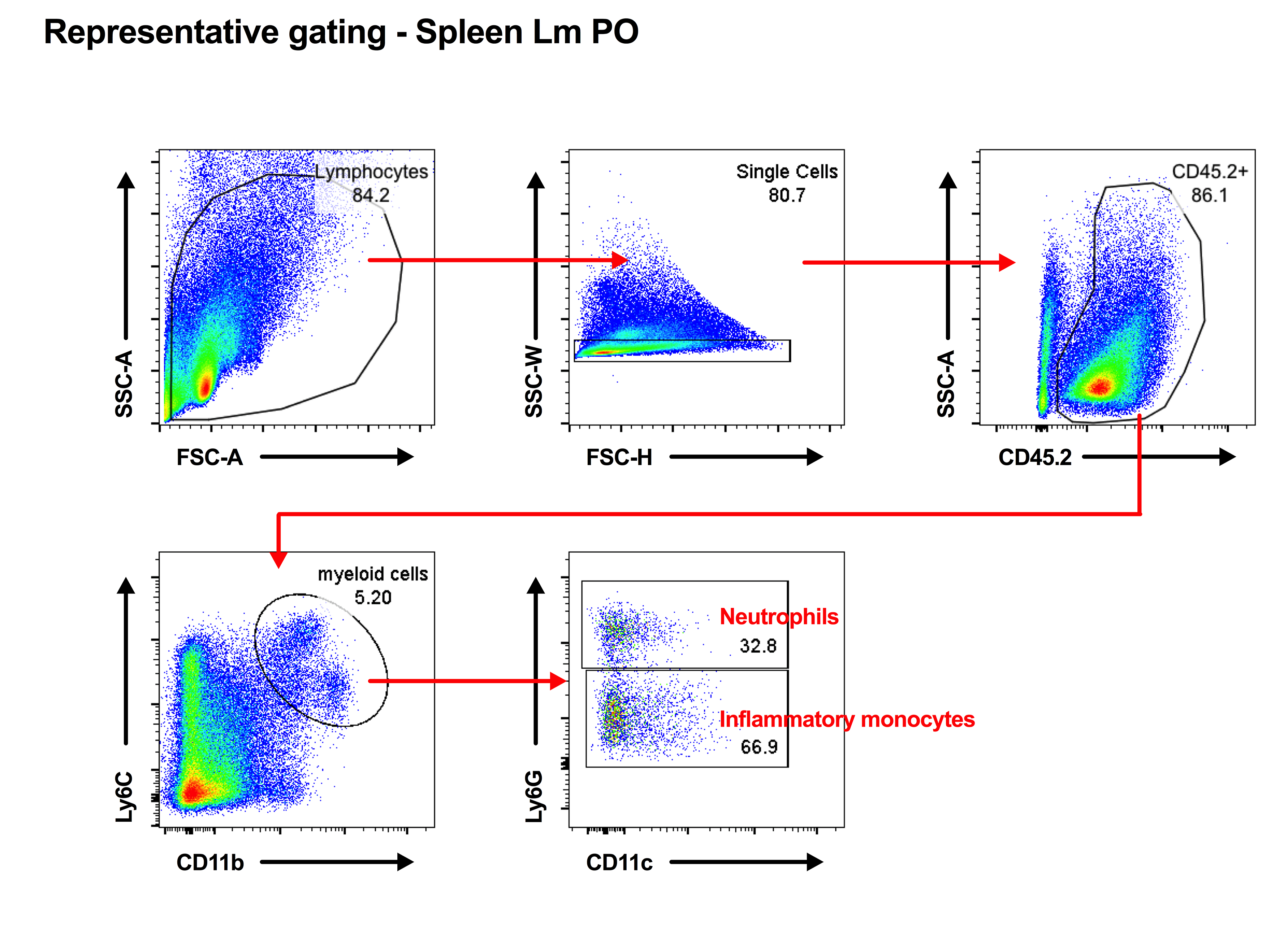

Supplement: S5 Fig — Representative flow cytometry plots from the spleen of a B6.tiger (Il10-gfp reporter) mouse infected with Lm PO and harvested at 3 dpi. (TIF) [file ppat.1009531.s005.tif]

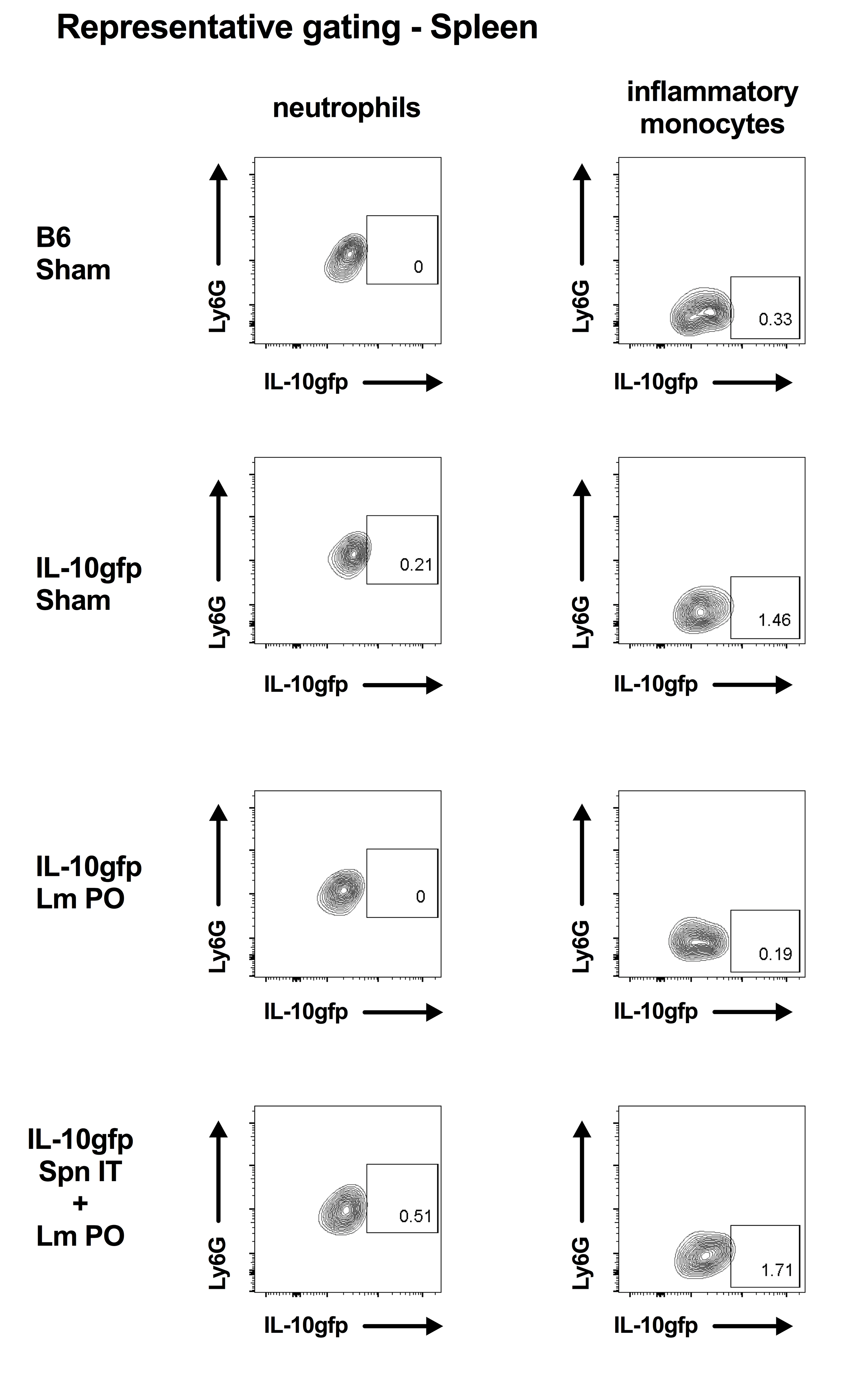

Supplement: S6 Fig — Representative flow cytometry plots from the spleens of sham infected control B6 mice and B6.tiger (Il10-gfp reporter) mice infected with sham control, Lm PO, or Spn IT + Lm PO and harvested at 3 dpi. (TIF) [file ppat.1009531.s006.tif]

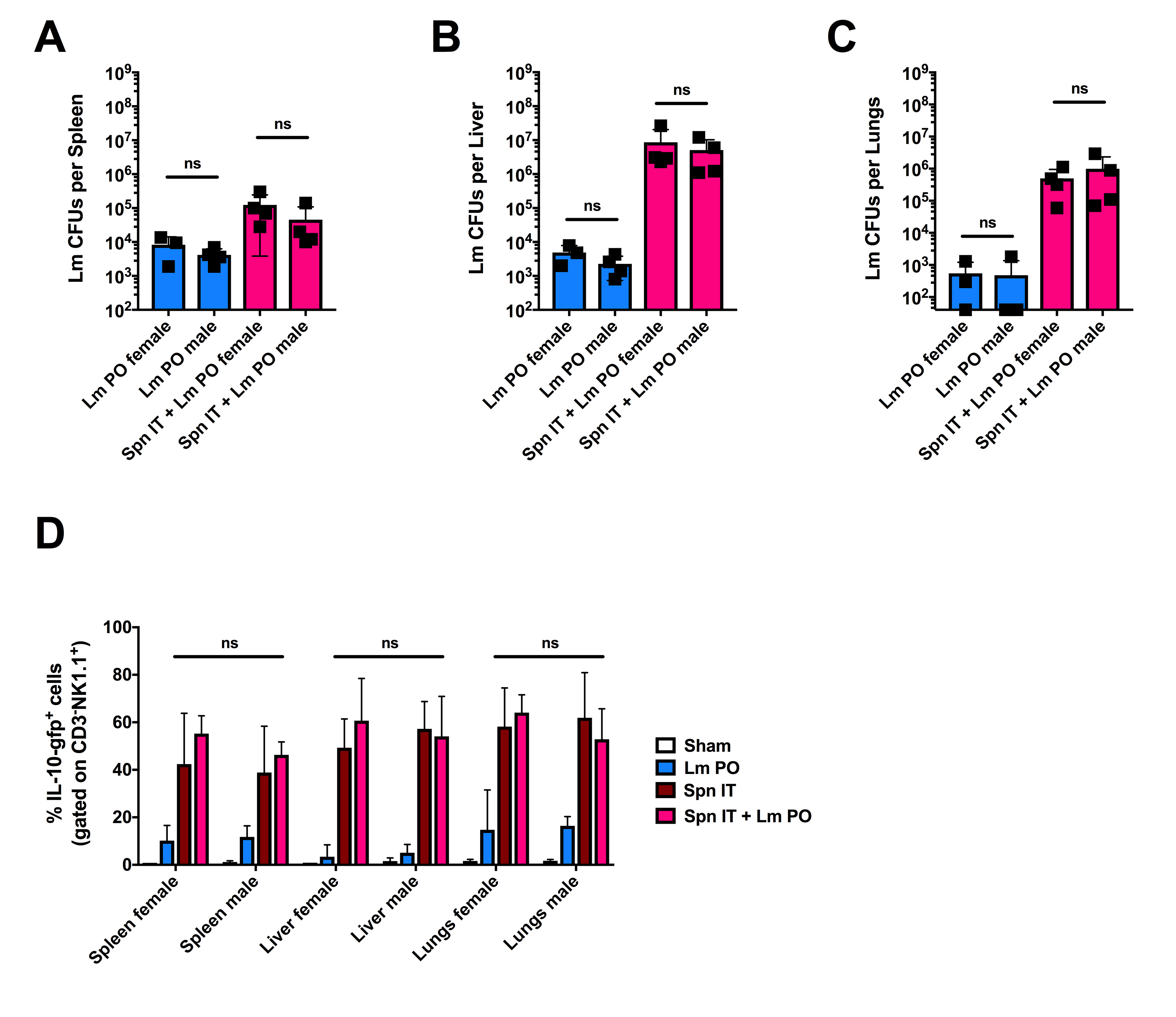

Supplement: S7 Fig — B6.tiger (Il10-gfp reporter) male and female age-matched mice were infected with sham control, Lm PO, Spn IT, or Spn IT + Lm PO and harvested at 3 dpi. Lm burdens were enumerated from (A) spleens, (B) livers, and (C) lungs. Data represent mean ± SD, Mann-Whitney t-test, pooled from 2 experiments, 3–4 mice per group. (D) Quantitation of percent CD3-NK1.1+ NK cells that stained positive for IL-10-gfp+ in spleens, livers, and lungs. Data represent mean ± SD, Two-way ANOVA, pooled from 2 experiments, 3–4 mice per group. (TIF) [file ppat.1009531.s007.tif]

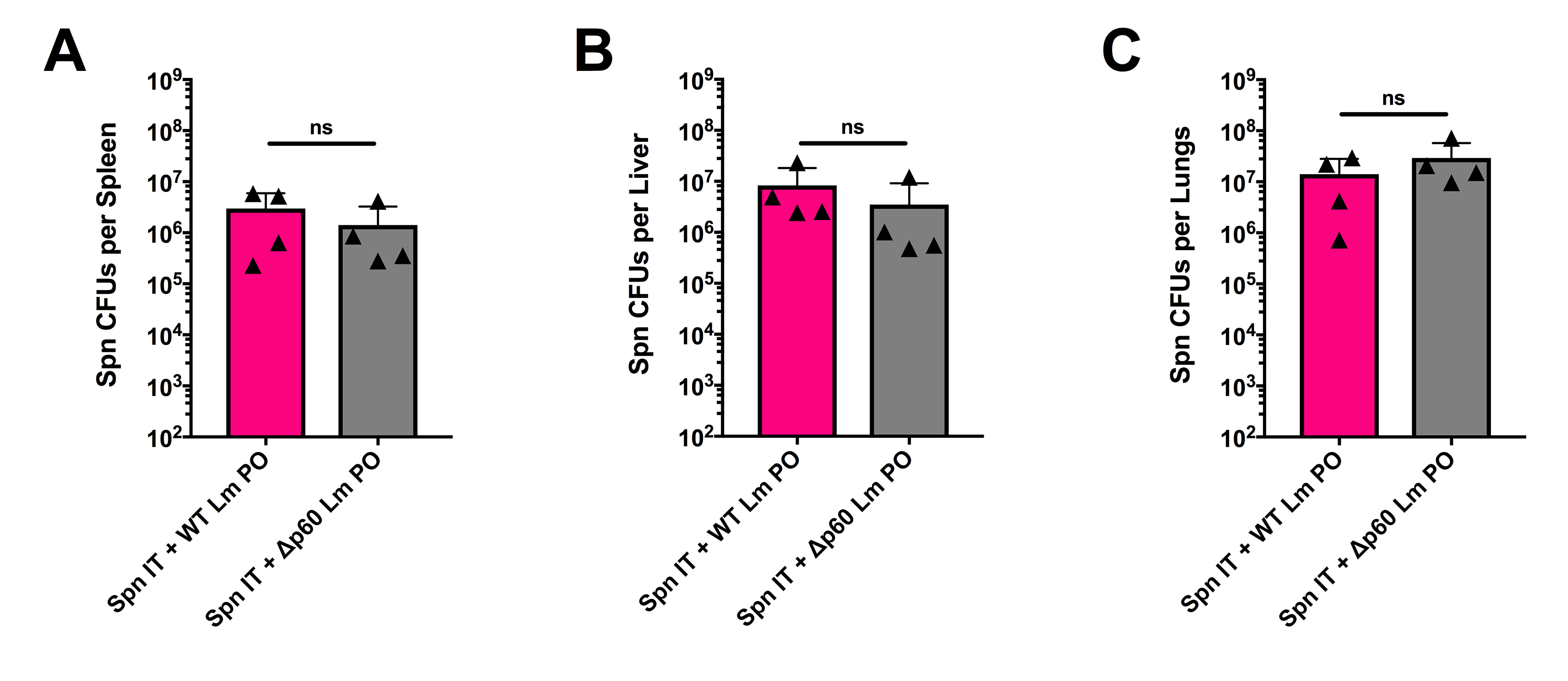

Supplement: S8 Fig — Spn burdens from mice in Fig 3E, 3F, and 3G were enumerated in (A) spleens, (B) livers, (C) lungs. Data represent mean ± SD, Mann-Whitney t-test, pooled from 2 experiments, 4 mice per group. (TIF) [file ppat.1009531.s008.tif]

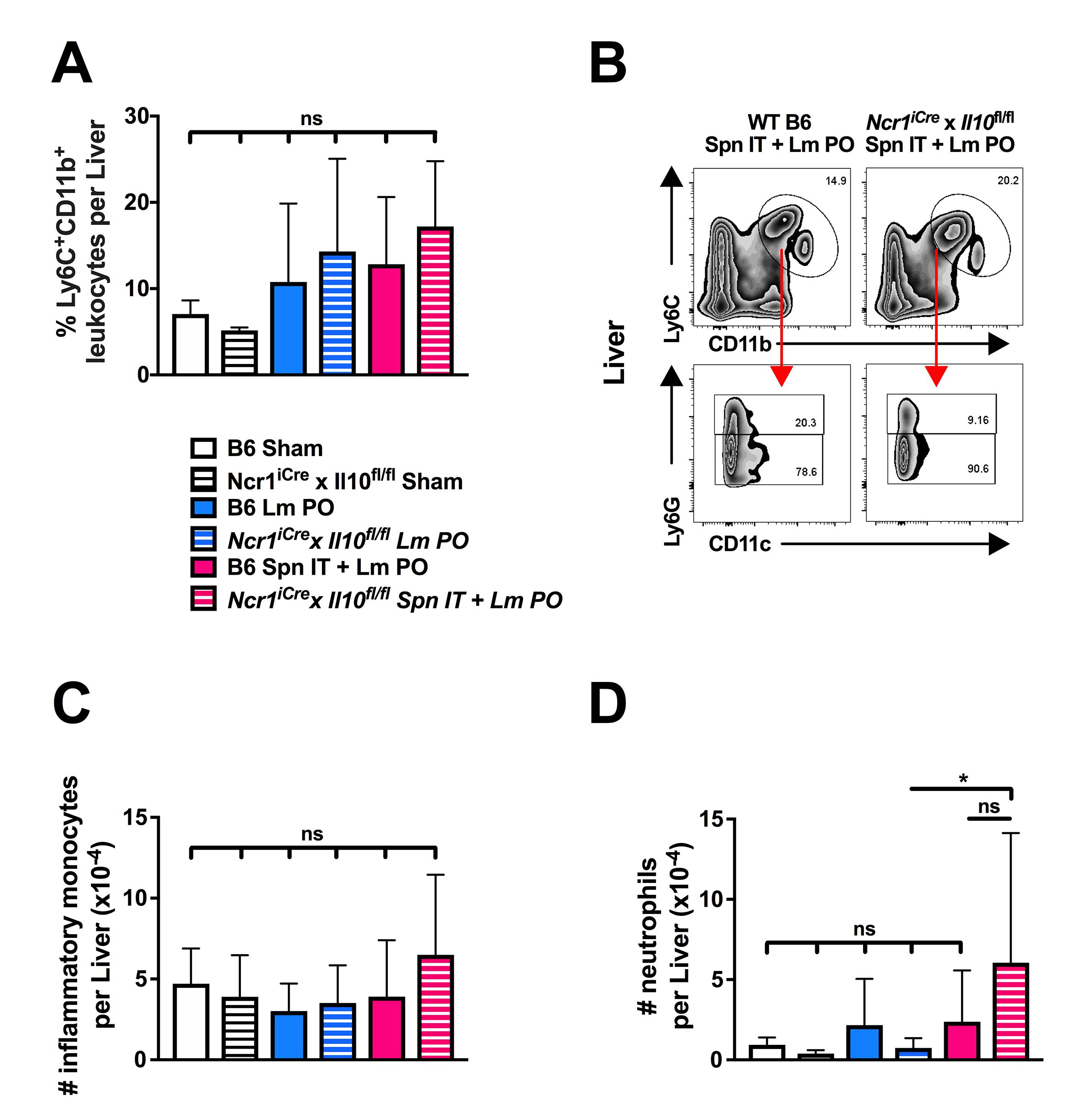

Supplement: S9 Fig — C57BL/6J (B6) and Ncr1iCrexIl10fl/fl male and female age-matched mice were infected with sham control, Lm PO, or Spn IT + Lm PO and livers were harvested at 3 dpi. (A) Quantitation of percent CD45.2+Ly6C+CD11b+ leukocytes. Data represent mean ± SD, One-way ANOVA, pooled from 3 experiments, 6–12 mice per group. Representative flow plots of Ly6G+ neutrophils and Ly6G-CD11cl° inflammatory monocytes in B6 and Ncr1iCrexIl10fl/fl coinfected mice. Quantitation of total number of CD45.2+Ly6C+CD11b+ cells that are Ly6G-CD11cl° inflammatory monocytes (C) or Ly6G+ neutrophils (D). Data represent mean ± SD, One-way ANOVA, pooled from 3 experiments, 6–12 mice per group, *p<0.05 between indicated groups. (TIF) [file ppat.1009531.s009.tif]

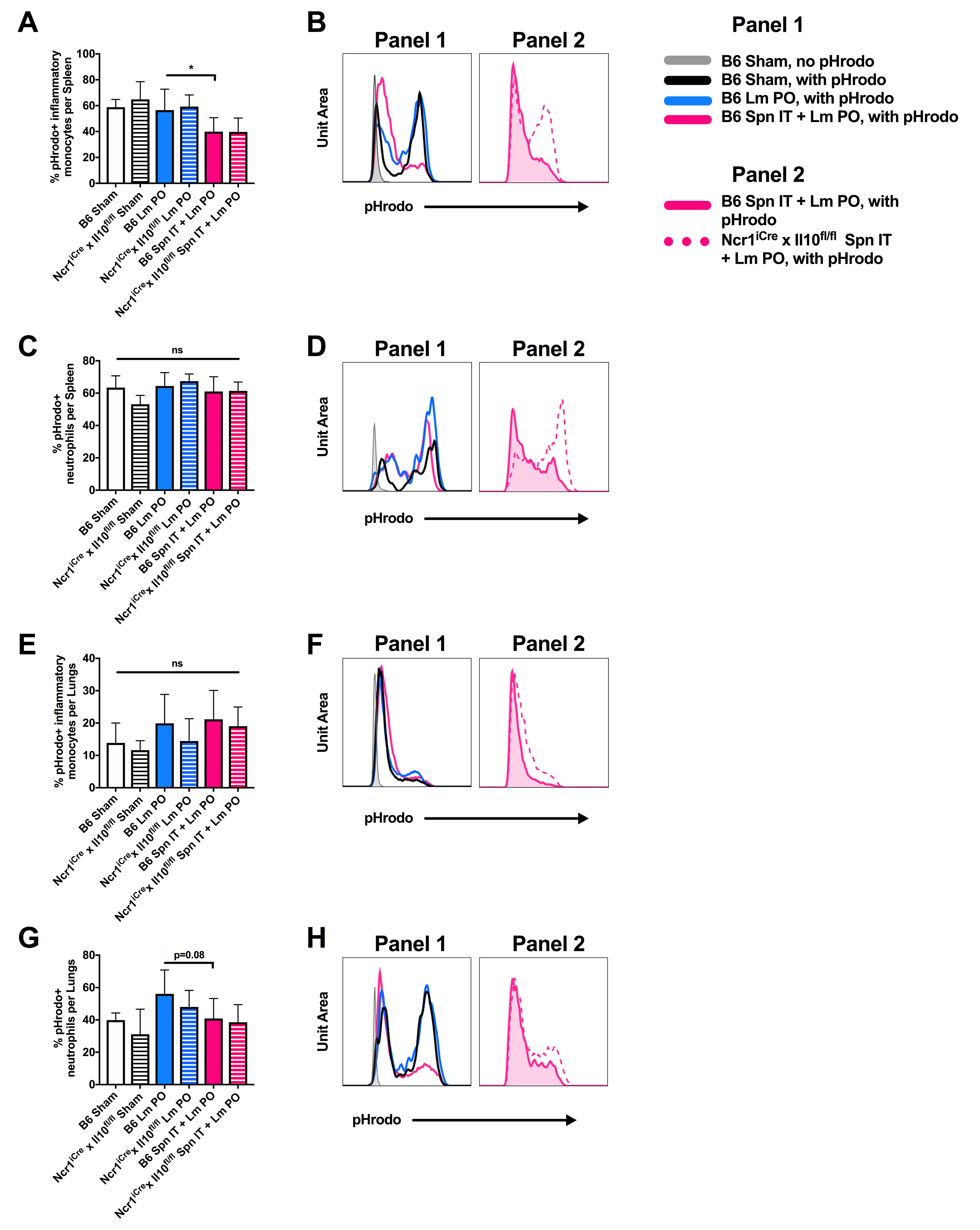

Supplement: S10 Fig — pHrodo S. aureus Bioparticles were incubated for 1 hr with cells isolated from spleens and lungs. Percent pHrodo positive inflammatory monocytes and neutrophils were determined by flow cytometry. %pHrodo+ from inflammatory monocytes in spleens (A), neutrophils in spleens (C), inflammatory monocytes in lungs (E), and neutrophils in lungs (G). Data represent mean ± SD, One-way ANOVA, pooled from 2 experiments, 8–9 mice per group, *p<0.05 between indicated groups. Representative pHrodo staining from inflammatory monocytes in spleens (B), neutrophils in spleens (D), inflammatory monocytes in lungs (F), and neutrophils in lungs (H). In panel 1: Light gray shaded line represents Sham infected B6 mice with no pHrodo staining, Black line represents Sham infected B6 mice with pHrodo staining, Blue line represents Lm PO infected B6 mice with pHrodo staining, Pink line represents Spn IT + Lm PO infected B6 mice with pHrodo staining. In panel 2: Pink shaded line represents Spn IT + Lm PO infected B6 mice with pHrodo staining, while Pink dotted line represents Spn IT + Lm PO infected Ncr1iCrexIl10fl/fl mice with pHrodo staining. (TIF) [file ppat.1009531.s010.tif]

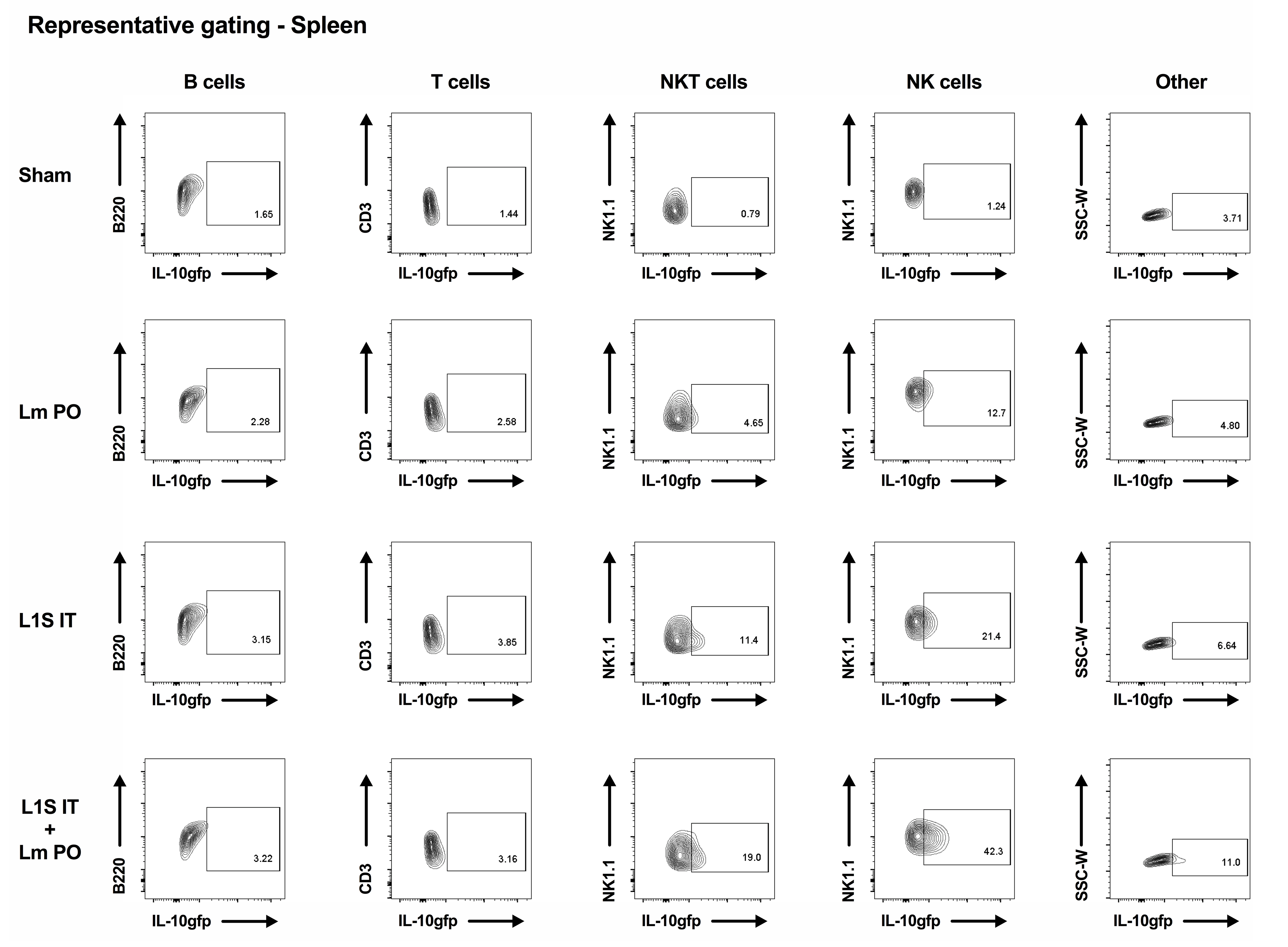

Supplement: S11 Fig — Representative flow cytometry plots from the spleens of B6.tiger (Il10-gfp reporter) mice infected with sham control, Lm PO, L1S IT, or L1S IT + Lm PO and harvested at 3 dpi. (TIF) [file ppat.1009531.s011.tif]

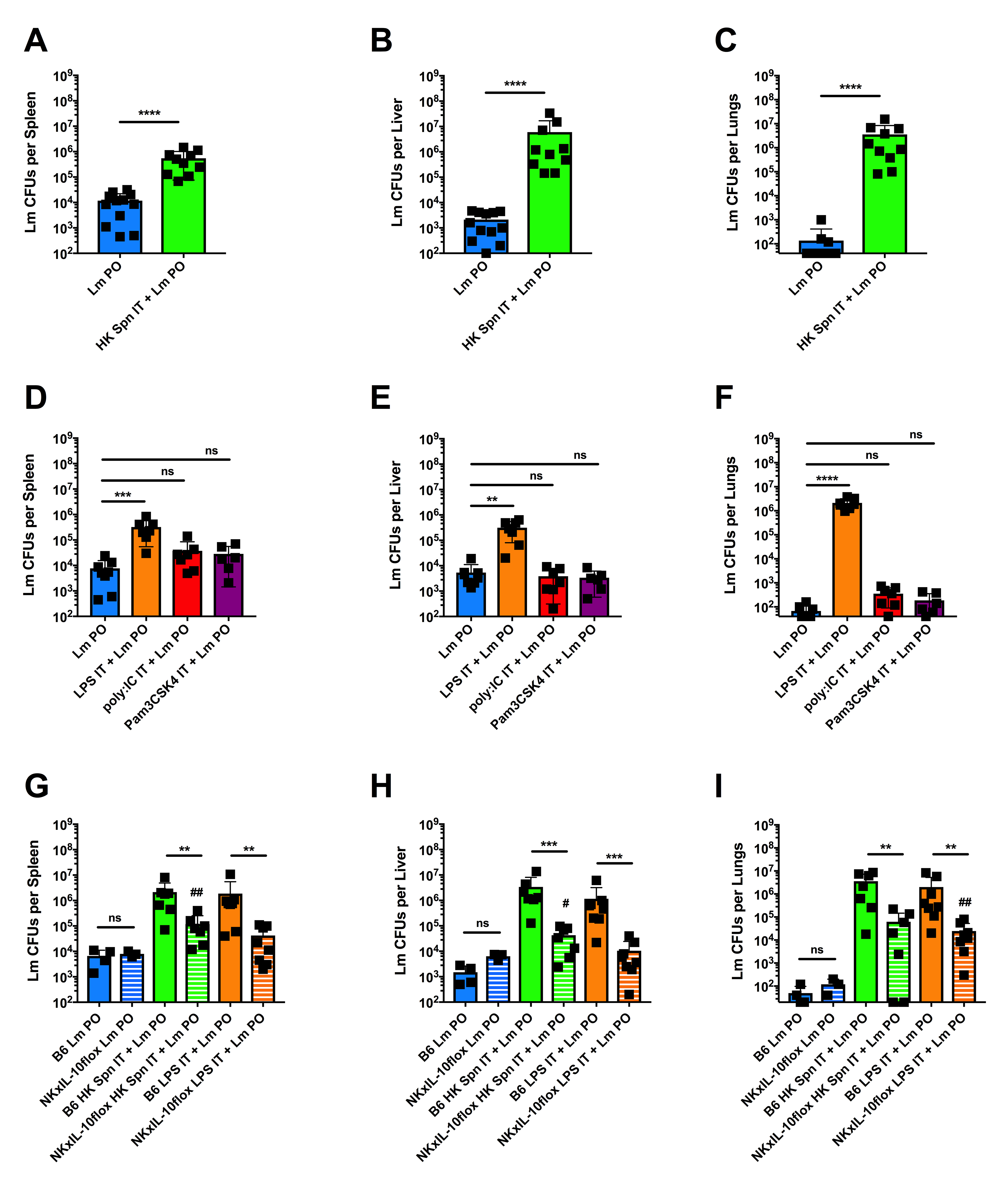

Supplement: S12 Fig — C57BL/6J (B6) male and female age-matched mice were infected with Lm PO or 106 HK Spn IT + Lm PO, and CFUs were determined 3 dpi. Lm burdens were enumerated from (A) spleens, (B) livers, and (C) lungs. Data are represented as mean ± SD, Mann-Whitney t-test, pooled from 3 experiments, with 10–12 mice per group, ****p<0.0001 between indicated groups. B6 male and female age-matched mice were infected with Lm PO, or co-treated with 50ug LPS IT + Lm PO, 50ug poly:IC IT + Lm PO, or 50ug Pam3CSK4 IT + Lm PO and CFUs were determined 3 dpi. Lm burdens were enumerated from (D) spleens, (E) livers, and (F) lungs. Data are represented as mean ± SD, Mann-Whitney t-test, pooled from 2 experiments, with 8–10 mice per group, **p<0.002, ***p<0.0002, ****p<0.0001 between indicated groups. C57BL/6J (B6) and Ncr1iCrexIl10fl/fl male and female age-matched mice were infected with Lm PO, 106 HK Spn IT + Lm PO, or 50ug LPS IT + Lm PO and harvested at 3 dpi. Lm burdens were enumerated from (G) spleens, (H) livers, and (I) lungs. Data represent mean ± SD, Mann-Whitney t-test, pooled from 2 experiments, 4–8 mice per group. **p<0.002, ***p<0.0002, between indicated groups, #p<0.05, ##p<0.001 between indicated co-treated Ncr1iCrexIl10fl/f mice and B6 Lm PO infected mice. (TIF) [file ppat.1009531.s012.tif]
